# Management and outcome of children with high-risk neuroblastoma: insights from the Spanish Society of Pediatric Hematology and Oncology (SEHOP) neuroblastoma group on refractory and relapse/progressive disease

**DOI:** 10.1007/s12094-025-03853-w

**Published:** 2025-02-25

**Authors:** Blanca Martínez de las Heras, Pedro M Rubio-Aparicio, Alba Rubio-San-Simón, Lucas Moreno, Paula Mazorra, Ricardo López Almaraz, Mercedes Llempén López, Julia Balaguer Guill, Vanessa Segura, Mar Bermúdez, Irene Jiménez, Désirée Ramal, Adela Cañete

**Affiliations:** 1 Pediatric Hemato-Oncology Department, Hospital Universitario y Politécnico La Fe, European Reference Network PAEDCAN member, Valencia, Spain; 2 Pediatric Hemato-Oncology Department, Hospital Universitario La Paz, European Reference Network PAEDCAN member, Madrid, Spain; 3 Pediatric Hemato-Oncology Department, Hospital Infantil Universitario Niño Jesús, Madrid, Spain; 4 Pediatric Department, Hospital Universitario Vall d´Hebron, European Reference Network PAEDCAN member, Barcelona, Spain; 5 Pediatric Onco-Hematology Unit, Hospital Cruces, and Pediatric Oncology Group Biobizkaia Health Research Institute, Barakaldo, Spain; 6 Pediatric Oncology Department, Hospital Universitario Virgen del Rocío, European Reference Network PAEDCAN member, Sevilla, Spain; 7 Clinical and Translational Oncology Research Group, Investigation Institute La Fe, Valencia, Spain; 8 Pediatric Hematology-Oncology Department, Hospital Clínico Universitario Virgen de la Arrixaca, Murcia, Spain

**Keywords:** Neuroblastoma, Relapse, Immunotherapy, Clinical trials

## Abstract

**Purpose:**

Outcome for children with refractory and relapse/progressive high-risk neuroblastoma (HR-NB) remains poor, without an internationally agreed standard second-line approach. Heterogeneity in patients’ disease and treatment strategies challenges clinical management. The survival rate for patients with resistant disease does not exceed 20% at 5 years. The study’s aim was to analyze refractory and progressive HR-NB patients in a real-world setting to evaluate current clinical practices and optimize future approaches.

**Methods:**

Data from patients diagnosed with refractory and relapse/progressive (R/R-P) HR-NB between January 2019 and December 2021 at six of the major Spanish neuroblastoma treating hospitals were collected and analyzed.

**Results:**

A total of 67 episodes of R/R-P HR-NB were included. Treatments applied included chemotherapy (97%), immunotherapy (48%), consolidation (21%), local treatment (surgery and/or radiotherapy) (45%) and maintenance (16%), and were administered within a clinical trial (CT) in 34% of the episodes. Biopsy was performed in 37% of the tumors and 30% were profiled. Event-free survival (EFS) in our cohort was 20.9% and overall survival (OS) 32%. Significant survival advantage (in both OS and EFS) was observed in refractory episodes compared to relapse/progressive, in first events compared to successive, and when response or disease stabilization was achieved. *MYCN* status, presence of lymph node metastases, use of irinotecan or topotecan, and radiotherapy were also univariate predictors of OS.

**Conclusions:**

Treatment of refractory and relapse/progressive HR-NB is highly heterogeneous. We confirm a poor outcome, although certain epidemiological and treatment-related factors have prognostic value. Molecular profiling and inclusion in CTs should be improved.

**Supplementary Information:**

The online version contains supplementary material available at 10.1007/s12094-025-03853-w.

## Introduction

High-risk neuroblastoma (HR-NB) accounts for up to 50% of the neuroblastic tumor diagnoses [1]. The International Neuroblastoma Risk Group Staging System (INRGSS) defines HR-NB by the presence of metastatic disease (stage M) in patients diagnosed above 12 months of age; or by the amplification of MYCN (MNA), in unresectable or metastatic tumors (stage L2, Ms and M) [2]. The most frequent sites of metastasis are bone marrow (80%) and cortical bone (60%) [3]. In 40% of the HR-NB patients, the tumor presents with MNA [4], associating worse prognosis [5–7].

Although multimodal therapy has increased overall survival (OS) from 15 to 50% in the last 50–60 years [8, 9], outcome is still fatal in almost half of the patients with HR-NB [5, 7], due to lack of response to first-line treatment or recurrence after or during treatment, with little chance for cure [10, 11].

Efforts now focus on tailoring treatment, using response biomarkers and prognostic factors in both first-line and refractory and relapse/progressive (R-R/P) scenarios with immunotherapy and targeted agents leading the way. Unlike first-line HR-NB, R/R-P disease lacks a standardized approach, making management heterogeneous and challenging.

The Spanish Society of Pediatric Hematology and Oncology (SEHOP) Neuroblastoma Working Group, established in 1987, aims to improve survival in Spanish children with neuroblastoma through national collaboration. According to the Spanish Registry of Children with Tumors (RETI-SEHOP) and Cañete et al. (2022), Spain reports 80–90 cases NB cases annually in children aged 0–14, including approximately 45 HR-NB and 20 R/P cases per year [12, 13].

In this paper, we analyze the standard of care in R/R-P HR-NB, focusing on clinical management and prognosis, in a recent cohort from our country. This study also aims to evaluate current practices in order to identify opportunities for improvement.

## Methods

### Study design and patients

In this retrospective, multicentric study, we collected information from patients with HR-NB at the time of relapse, progression or whose disease was refractory to first line between 1st January 2019 and 31st December 2021, from 6 highly recruiting hospitals in Spain. Hospital selection was based on neuroblastoma accrual, accreditation as reference center by National Certification (Reference Unit of the National Health System, CSUR) or by number of second opinion requests from other centers. Ethics Committee approval was obtained prior to start data collection.

Inclusion criteria were: HR-NB according to the International Society of Paediatric Oncology European Neuroblastoma (SIOPEN) modified International Neuroblastoma Risk Group (INRG) criteria: stage M neuroblastoma above 365 days of age at diagnosis; or MNA and INRG stage above L1 regardless of age; and confirmed refractory, relapsed or progressive disease. Patients were allowed to enter more than once in the study, if they experienced successive R/R-P events.

A data collection form was sent to each center in order to be filled out through an individual patient record review. Clinical and biologic data collection of the initial diagnosis for each patient included age at diagnosis, sex, INRGSS stage, front-line treatment, *MYCN* status and other genetic studies.

The following data were recorded from each episode: date and disease status (either relapse/progression or refractory disease), first or successive event, tumor biopsy, tumor genetic profiling, inclusion in a CT, treatment received, response to treatment according to International Neuroblastoma Response Criteria (INRC), outcome (OS and EFS), and follow-up with 31 December 2022 as cut-off date.

Data on second opinion referrals were gathered with local oncologists help.

Events leading to enrollment were categorized as refractory and relapse/progression. Relapse and progressive events were analyzed together as progressions, following the consensus proposal from the National Cancer Institute (NCI) [14]. Refractory disease was defined as incomplete response of HR-NB to front-line treatment (at least 4 cycles of induction), without new lesions nor increase of disease burden. Progression was considered, per INRC definition [15], as appearance of new lesions, or objective increase of known lesions. Subsequent events in the same patient were considered as relapses/progressions during the study period.

### Statistical methods

Descriptive statistics were reported as absolute frequencies and percentages for qualitative data and for numerical variables median and interquartile range (IQR) were used. Differences in variable frequencies were evaluated using the Chi-square or Fisher’s exact test, as appropriate.

Objective response rate (ORR) was calculated at the end of complete salvage treatment as complete response (CR), partial response (PR), or stable disease (SD), which included minor response (MR) and minimal disease (MD), or when subsequent progressive event occurred as progressive disease (PD) according to INRC. Responders reached CR or PR, and clinical benefit included also SD.

Reported responses correspond to best response as a whole to the rescue treatment received.

OS was evaluated from study entry, considered as the date of event (relapse/progression or refractory disease), to death or date of last follow-up in survivors, by the Kaplan–Meier method, and differences between groups were assessed by the log-rank test. When OS is less than 50%, we refer to median survival time. We also calculated the EFS, defined as the time from any event of refractory or relapse/progression disease to further progression, death from any cause, or date of last follow-up. The effect of the analyzed prognostic factors was assessed by the Cox regression model. All statistical tests were two-sided and a *p* value less than 0.05 was considered significant. All analyses were performed using the IBM SPSS statistics version 25.

## Results

### Patient characteristics

We included 49 patients with HR-NB accounting for a total of 67 episodes (7 episodes of refractory disease and 60 of relapse/progression). Median age at time of study entry was 62.5 months (IQR = 51–93.5). Half of the episodes (*n* = 33) were referrals from other centers for second opinion on therapeutic management decision. From 33 referrals, 19 received treatment in hospitals different from their own.

Relevant patients’ characteristics are shown in Table 1. Most of the cases were stage M (91.8%) at first diagnosis and the rest were L2. Median time from first diagnosis to the event of study was 14 months (IQR = 6.5–30.5), and from the end of the previous treatment to the event was 0 (IQR = 0–4). At R/R-P episode, 89.5% of the patients had metastatic involvement (79.2% only metastatic; 10.4% combined with local involvement), and the remaining 10.4% were localized. Among the metastatic patients, 55.4% affected more than one compartment. Bone (81%) and bone marrow (35.6%) were the most frequently involved.

**Table 1:**
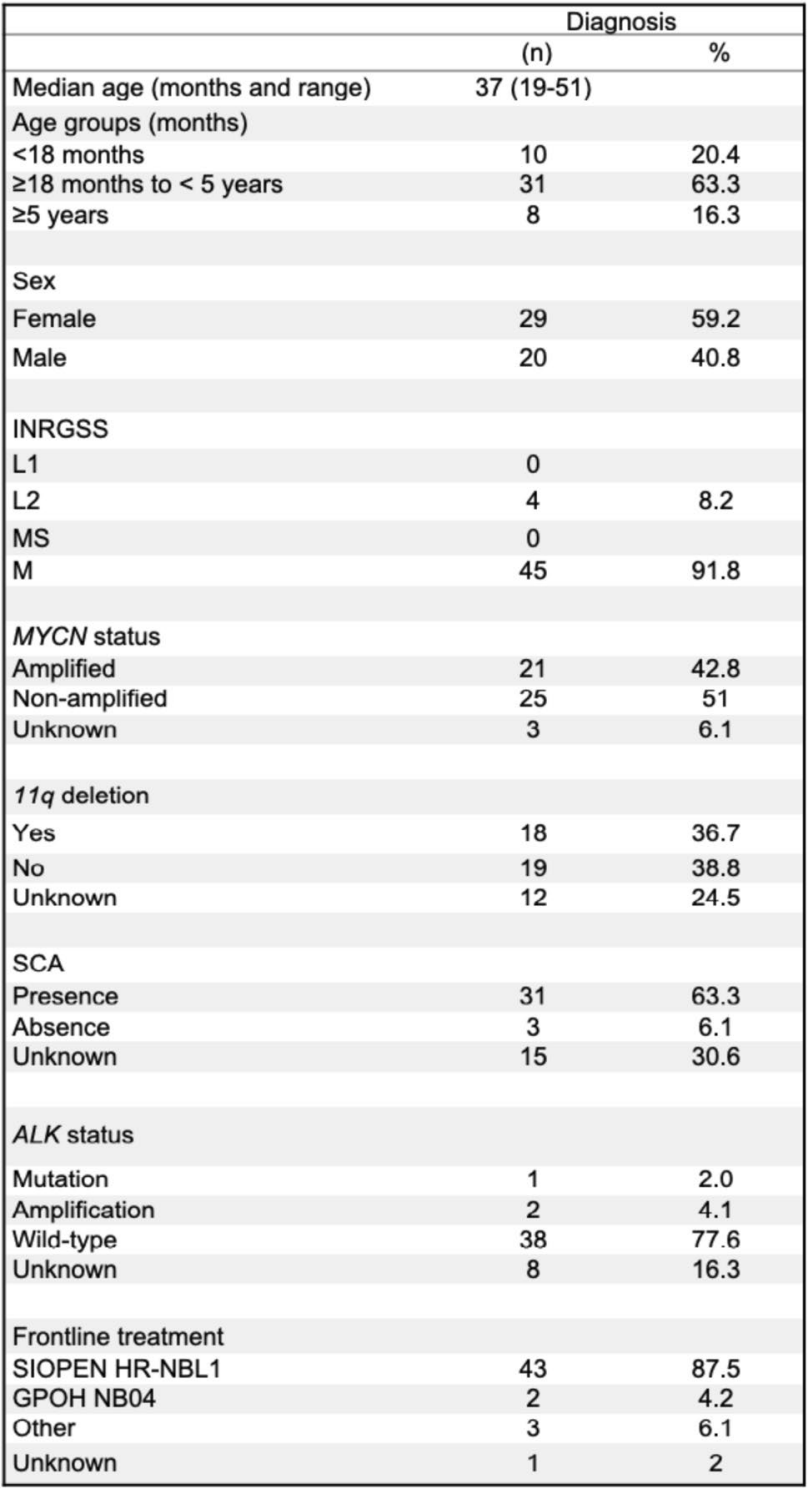
Patient characteristics at diagnosis (*n* = 49)

*INRGSS* International Neuroblastoma Risk Group Staging System, *SCA* segmental chromosomal aberrations

For 65.7% of the episodes registered (*n* = 44), it was the first event. For the remaining 34.3% (*n* = 23), it was a recurring event (second for 14 episodes, third for 6, and fourth for 3) (Table 2).

**Table 2:**
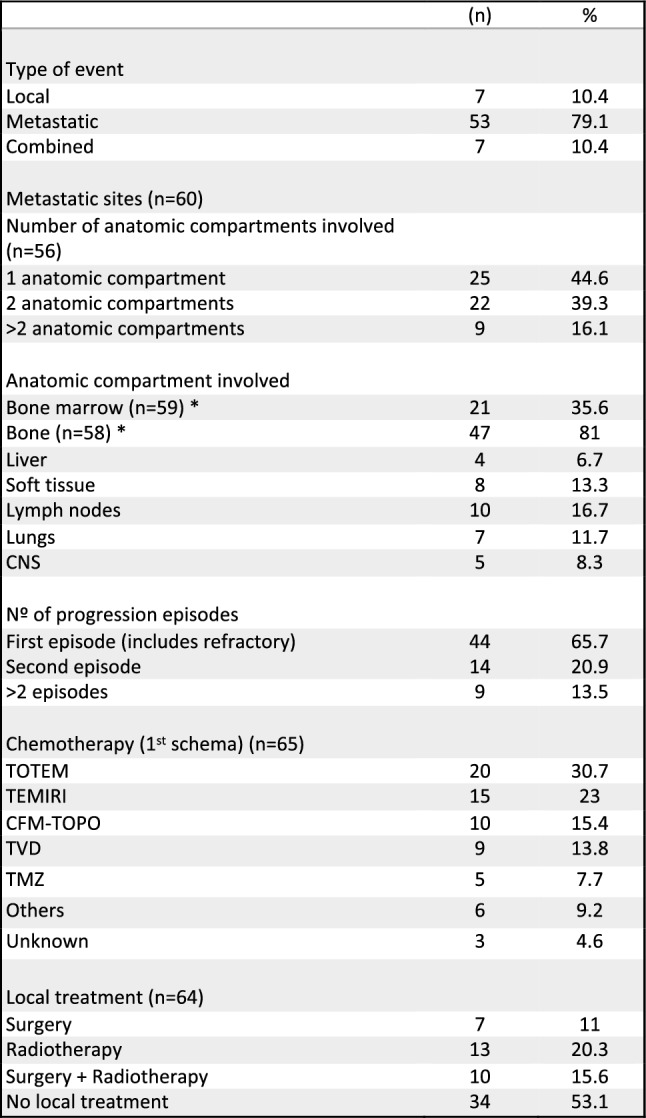
Characteristics and treatment data of the total events of refractory and relapsed/progressive disease (*n* = 67)

*TOTEM* topotecan–temozolomide, *TEMIRI* irinotecan–temozolomide, *CFM-TOPO* cyclophosphamide–topotecan, *TVD* topotecan, vincristine and doxorubicin, *TMZ* temozolomide, *CNS* central nervous system

*Unknown data (1 episode for bone marrow involvement and 2 episodes for bone involvement)

### Management of R/R-P episodes

Twenty-five biopsies (37.3%) were performed at study entry. We found no difference between first or recurring events (38.6% vs. 34.8%, *P* = *0.48*) nor between local or metastatic extension (42.9% vs. 35.8%, *P* = *0.426*). However, there was an observed trend towards increased biopsy rates among patients without bone involvement (55.6% vs. 31.9%, *P* = *0.072*). Next-generation sequencing (NGS) was performed in samples of 20 episodes (30%), although no relevant findings were reported.

Twenty-three episodes in 20 patients were treated in CTs: SIOPEN BEACON (NCT02308527) in 11 episodes; Erbumine (NCT04106219) in 3; Naxitamab (NCT03363373), AloCELYVIR (NCT04758533) and Omburtamab (NCT03275402) in 2 episodes each; CRISP (NCT00543753), SIOPEN VERITAS (NCT03165292) and Idasanutlin (NCT04029688) in one each.

Chemotherapy was administered to 65/67 episodes (97%). Regimens prescribed were mainly based in temozolomide (58.5%) and irinotecan/topotecan (72.3%) (Table 2). All survivors received one of the following chemotherapy schemes, principally as induction: topotecan–temozolomide (TOTEM), temozolomide–irinotecan (TEMIRI), topotecan–cyclophosphamide (CFM-TOPO) or topotecan–vincristine–doxorubicin (TVD). One case received upfront high-dose therapeutic 131I-mIBG and topotecan followed by autologous stem cell rescue (ASCR).

Immunotherapy was administered to 31 episodes (48%): anti-disialoganglioside-2 (anti-GD2) antibody in 27 (14 as induction combined with chemotherapy, 5 as consolidation, 7 as maintenance, 1 unknown), oncolytic virus in 2 as induction and 131I-Omburtamab in 2 as consolidation.

There were data of local treatment for 64 episodes. Surgery and/or radiotherapy (RT) were performed in 30 (46.8%): 10 episodes (15.6%) received both modalities, 7 (10.9%) only surgery and 13 (20.3%) only RT. 

Regarding consolidation, 14 (21%) received high-dose chemotherapy (HDC) with ASCR (7 busulfan-melphalan conditioning, 6 131I-mIBG, and 1 thiotepa conditioning). ASCR was received in 57% (*n* = 4) of refractory events vs. 17% relapse/progressive ones (*n* = 10) and it was associated to refractory and first relapse/progressive events (*P* = *0.032* and *P* = *0.016*, respectively) as 13 out of 14 ASCR performed was in a first event. Maintenance treatment was used in 11 patients (16.5%).

Details on treatment received per patient are available in supplementary material (Table S1).

### Antitumor response

For 66 evaluable patients (1 with no response data), response (CR and PR) was 24.3% (CR 18.2% + PR 6.1%) and clinical benefit (including SD) reached 35%. Sixty-five percent of the patients had PD.

Clinically relevant factors that showed association, in the univariate analysis, with a better ORR were absence of MNA (*P* = 0.002) and first episode (*P* = 0.006). Refractory patients might respond better than progressions (*P* = 0.053). In addition, the absence of ganglionar metastases associated with ORR (*P* = 0.049). Regarding treatment, use of temozolomide (*P* = 0.018) yielded better responses. Other treatments with favorable ORR were ASCR (*P* = 0.003) and radiotherapy (*P* = 0.002).

### Survival results

**Fig. 1:**
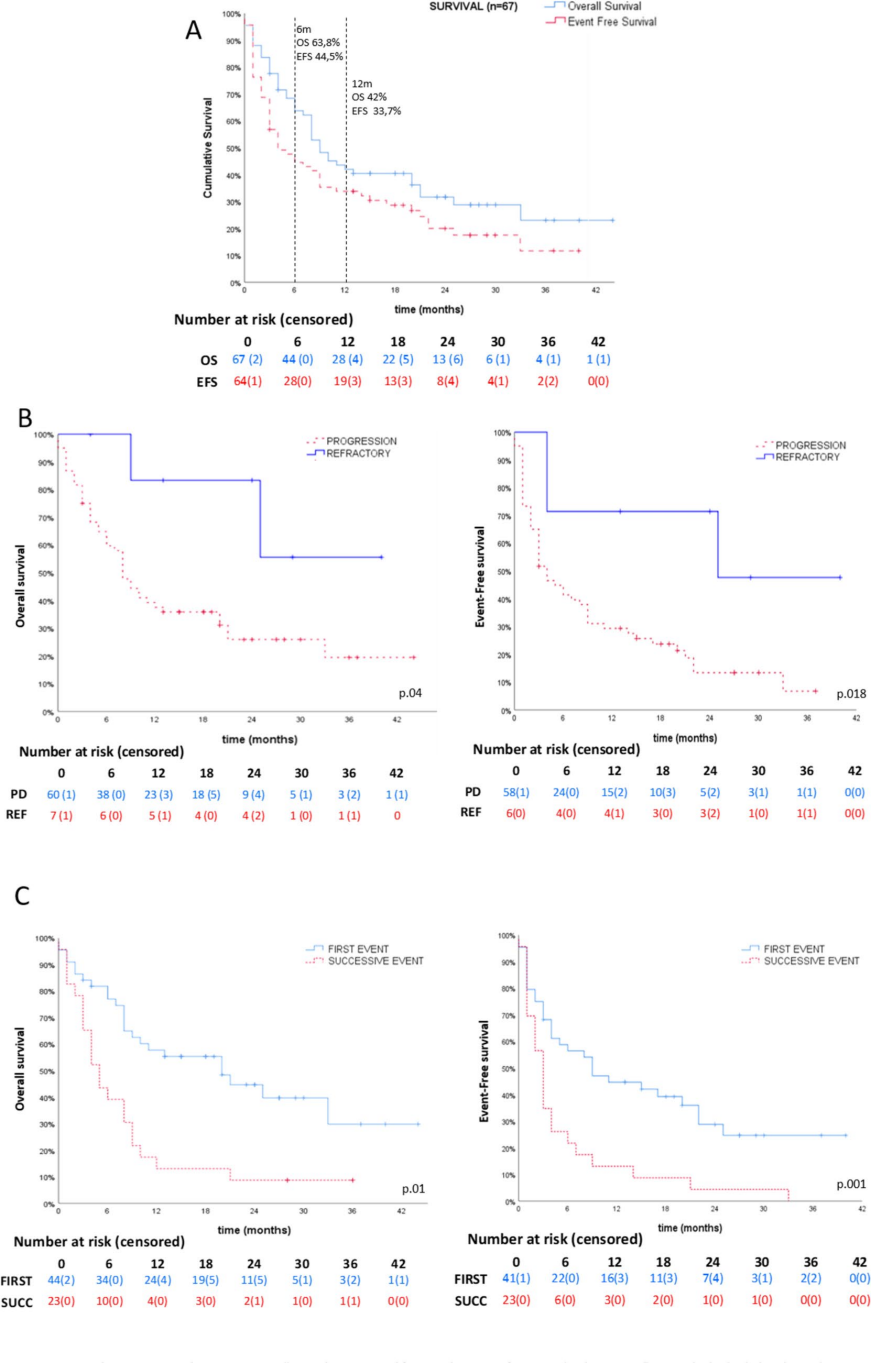
Kaplan–Meier OS and EFS curves. For all episodes, measured from study entry to first event (EFS), or exitus/last visit (OS). **A** Whole cohort (*n* = 67); **B** refractory (*n* = 7) vs. relapse/progressive episodes (*n* = 60); **C** first events (*n* = 44) compared to successive (*n* = 23). *OS* overall survival, *EFS* event-free survival

At the end of follow-up, OS in our cohort was 32%, with a median survival time of 9 months (IQR = 4–20, CI95 6.40–11.60). EFS was 20.9% with a median EFS time of 4 months (IQR = 2–17, CI95 1.06–6.94) (Fig. 1A).

There were statistically significant differences in OS and EFS when comparing refractory patients to progressive ones (OS 71.4% vs. 28.3%, *P* = 0.034; EFS 51.7% vs. 16.7%, *P* =  0.034), and in first events compared to the subsequent ones (OS 45.5% vs. 8.7%, *P* = 0.002; EFS 31.8% vs. 0%, *P* = 0.001) (Fig. 1B, C).

Treatment schemes including irinotecan/topotecan or temozolomide seemed to correlate with better survival (OS 39.6% vs. 15.8%, *P* = *0.053* and 47.7% vs. 13.8%, *P* = 0.003; *EFS* 29.2% vs. 0%, *P* =  0.005 and 28.9% vs. 10.3%, *P* = 0.058) but comparing TOTEM-TEMIRI against the rest of schemes (TVD, CFM-TOPO, VP-16 and carboplatin), we found no statistically significant difference (OS 42.4% vs. 27.6%, *P* = 0.171  and EFS 30.3% vs. 13.8%, *P* = 0.105).

**Fig. 2:**
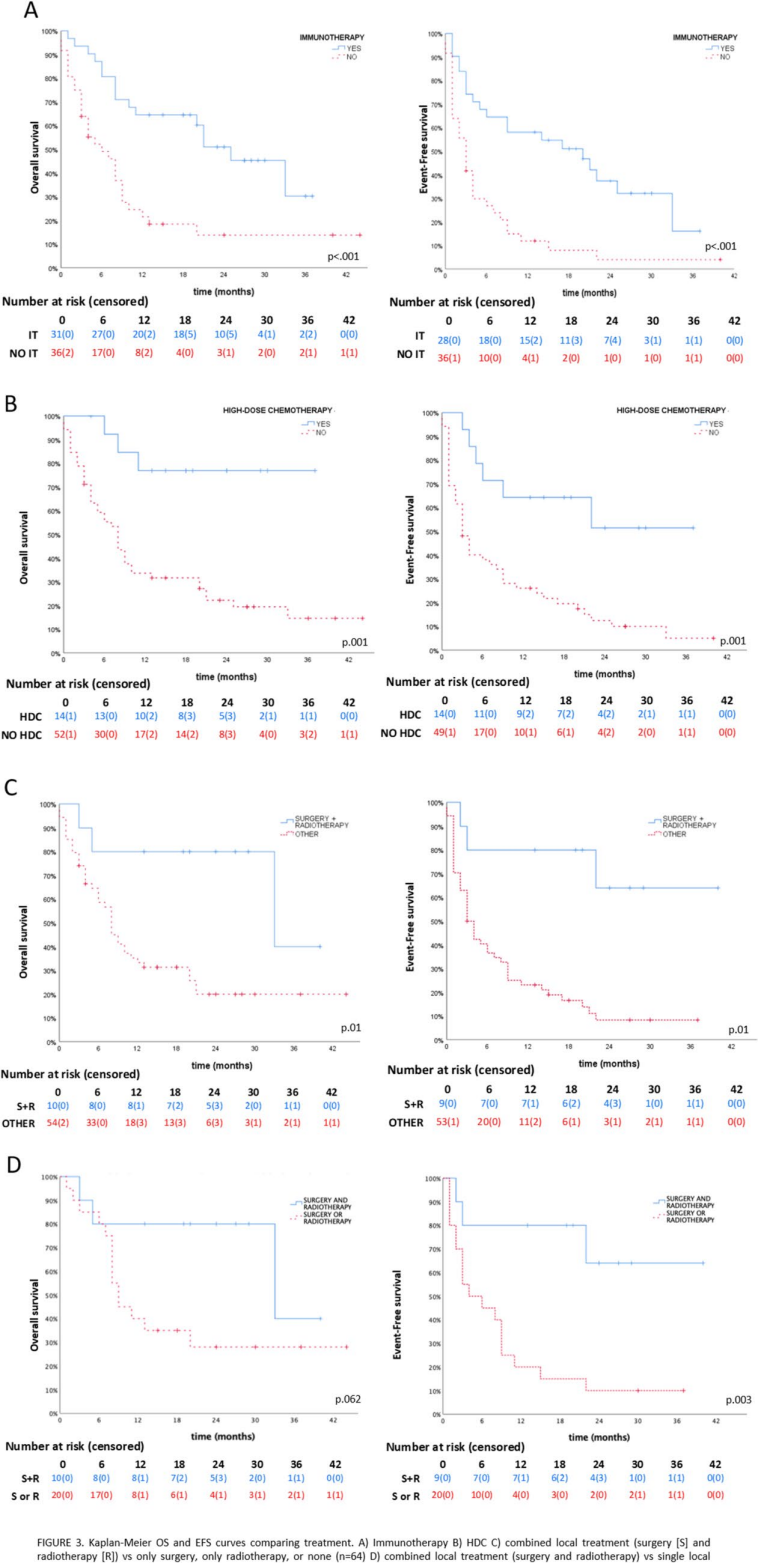
Kaplan–Meier OS and EFS curves comparing treatment. **A** Immunotherapy (*n* = 32), **B** HDC (*n* = 14), **C** combined local treatment (surgery and radiotherapy, *n* = 10) vs. only surgery (*n* = 7), only radiotherapy (*n* = 13), or none (*n* = 34). **D** Combined local treatment (surgery and radiotherapy, *n* = 10) vs. single local treatment (only surgery, *n* = 7, or only radiotherapy, *n* = 13), excluding cases with no local treatment (*n* = 34). In **C** and **D**, only patients with local or combined R/P are represented. OS: overall survival; *EFS* event-free survival;  *HDC* high-dose chemotherapy; *R/P* refractory/progression

**Fig. 3:**
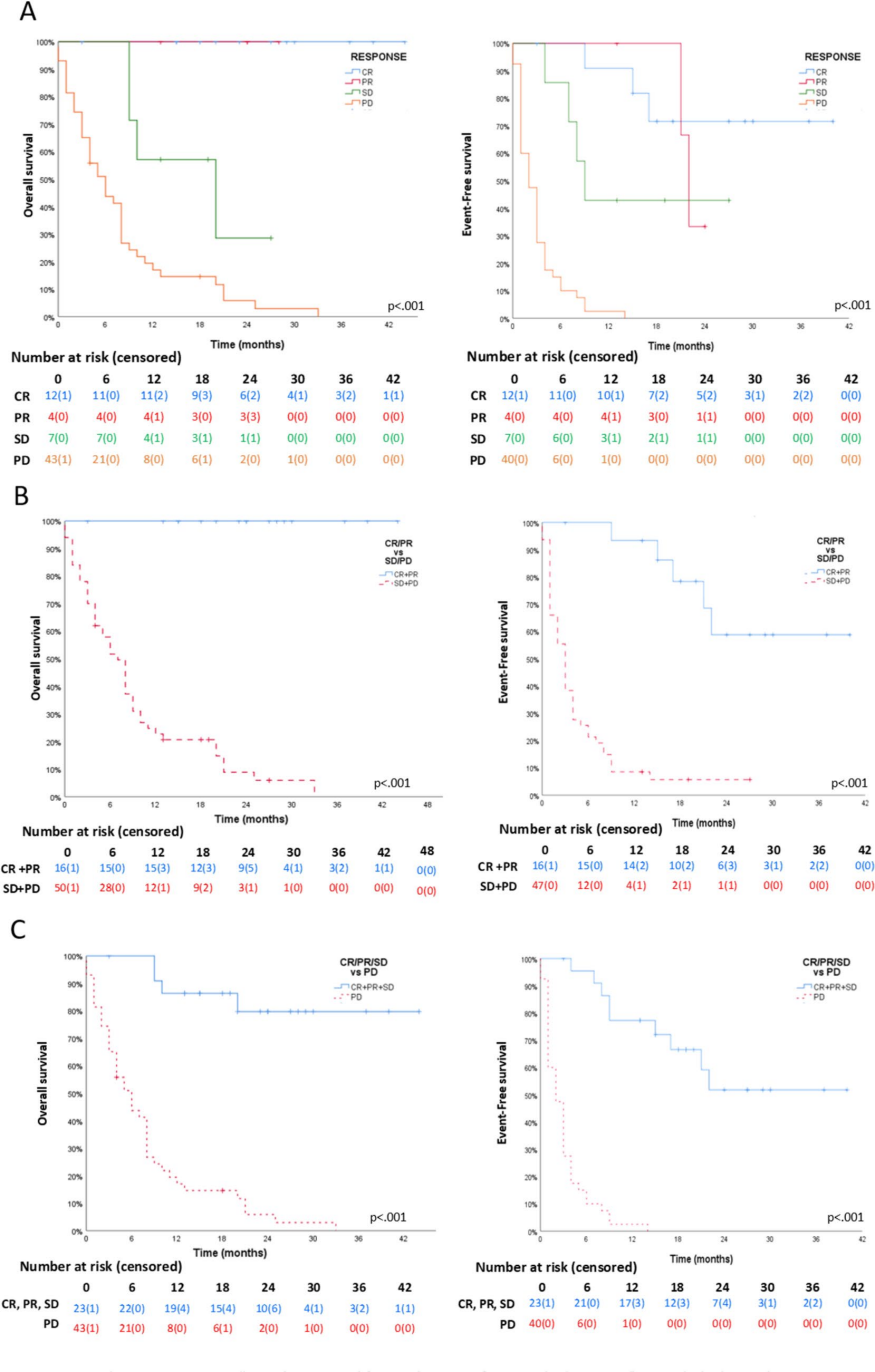
Kaplan–Meier curves. For all episodes, measured from study entry to first event (EFS), or exitus/last visit (OS). A OS and EFS curves according to the type of response (n = 66). B Responders (CR + PR, n = 16) vs. non-responders (SD + PD, n = 50). C Clinical benefit (CR + PR + SD, n = 23) vs. PD (n = 43). *CR* complete response, *PR* partial response, *SD* stable disease, *PD* progressive disease, *EFS* event-free survival, *OS* overall survival

Immunotherapy was associated with better outcome (OS 48.4% vs. 19.4%, *P* = 0.012; EFS 35.5% vs. 8.3%, *P* = 0.007) (Fig. 3A) as well as consolidation with ASCR (OS 78.6% vs. 21.2%, *P* < 0.001; EFS 57.1% vs. 11.5%, *P* = 0.001) (Fig. 2A, B).

Regarding episodes of local and combined progression, treatment with surgery + RT yielded better OS and EFS than the ones who did not receive any local treatment (OS 70% vs. 25.9%, *P* = 0.010 , EFS 70% vs. 13%, *P* = 0.001) or either only surgery or only RT excluding the ones who did not receive any local treatment (OS 70% vs. 30% *P* = 0.062 , EFS 70% vs. 10% *P* = 0.003) (Fig. 2C, D).

Responders had a higher and longer survival (*P* < 0.001): 100%/75% (OS/EFS) for CR; 100%/50% for PR. For SD, both OS and EFS were 42.9%. Patients with PD had an OS of 4.7%. Median survival time was not reached for CR (median follow-up was 25 months for OS and 19 months for EFS), nor for OS in PR (median follow-up 24 months). Median EFS for PR was 22 months. For SD, median survival was 20 months (OS) and 9 months (EFS). For PD, median survival was 8 months (OS) (Fig. 3). Complete survival analyses can be found in supplementary material (Figure S1).

### Multivariate analysis of prognostic factors

Multivariate analysis showed the following independent prognostic factors: for both OS and EFS, absence of MNA, use of irinotecan/topotecan and use of RT; for only OS, presence of lymph node metastases, and for EFS, use of local combined treatment (surgery and RT) (Table 3).

**Table 3:**
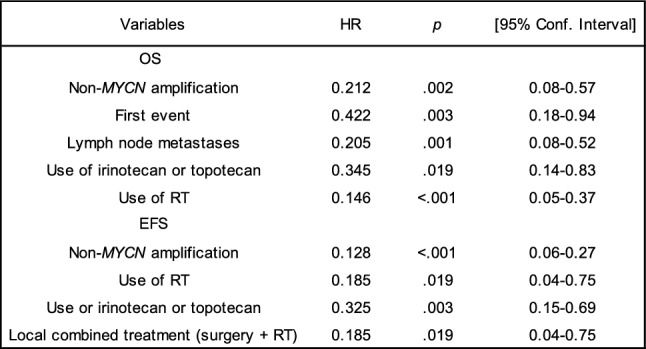
Multivariate analysis

Significative risk factors in R/R-P HR-NB

*HR* hazard ratio, *OS* overall survival, *RT* radiotherapy

## Discussion

In this retrospective review, we present an analysis of the demographic, biological, therapeutic and outcome data for all patients of R/R-P HR-NB diagnosed between 2019 and 2021 at six of the largest pediatric oncology centers in Spain. Our dataset comprises 7 patients of refractory neuroblastoma and 60 episodes of relapse/progression in 49 patients with HR-NB. Despite the advances in neuroblastoma treatment, our findings show that treatment choice is heterogeneous and survival rates remain unsatisfactory, supporting the need for internationally agreed consensus recommendations. These data offer a unique opportunity to discuss the management and outcomes of Spanish neuroblastoma patients within a representative cohort of real-world cases.

Several factors are used to assess risk stratification in neuroblastoma at the time of diagnosis, including molecular profiles [16, 17]. Our analysis revealed that almost all our patients exhibited molecular characteristics at first diagnosis (*n* = 49) associated with a high-risk of relapse, such as MNA (42.8%), *11q* deletion (36.7%), segmental chromosomal alterations (63.3%), and *ALK* alterations (6.1%). Despite the importance of the molecular studies for risk stratification and for providing efficient targeted therapy, *MYCN* status was not reported in 6.1% of our patients, and 30.6% did not have an available genomic profile.

Furthermore, only 37.3% (*n* = 25) of the patients were re-biopsied at event detection. Significant changes in molecular profile between diagnosis and progression are well documented in neuroblastoma [18, 19]; therefore, while prioritizing the patient’s well-being, it is strongly recommended to obtain tumor sample at the time of progression [20, 21]. Involving patient advocacy groups could play a crucial role by raising awareness among families about the importance of re-biopsy for accessing new therapies and CTs.

Time from diagnosis to first relapse has been identified as a prognostic factor [17, 22]. In our series, its median was 14 months, and 39% of the HR cases experienced relapse/progressive disease within 1 year from diagnosis. Median survival time from relapse/progression was 9 months, consistent with published data [17], despite the use of post-relapse newly strategies such as chemoimmunotherapy and new targeted therapies in many patients.

Patients with refractory disease demonstrated better OS compared to those with progressive disease [14], aligning with previously reported experiences [10, 23, 24]. These findings suggest that the management of R/R-P neuroblastoma in children may require distinct approaches. Several treatment variables were associated with outcome, particularly the use of immunotherapy, HDC with ASCR or combined local modalities.

Furthermore, any kind of response was also associated with significantly prolonged survival, which is all consistent with published data [25]. Multivariate analysis, although limited by sample size and heterogeneity, showed independent prognostic value for some treatment variables as the use of irinotecan/topotecan, RT and local combined treatment (surgery + RT) in addition to other clinical factors such as being a first episode of R/R-P disease, presence of metastasis in lymph nodes regardless of other affected sites or absence of MNA. Responders (CR and PR) had a 100% OS and 75% and 50% EFS, respectively. Patients that achieved SD also had a prolonged 20-month median survival. Nonetheless, the limited sample size and the considerable heterogeneity hinders drawing conclusions on all factors influencing outcomes.

The best management of R/R-P HR-NB patients remains to be established, and, consequently, treatment in daily clinical setting is challenging. Collaborative efforts are ongoing within SIOPEN to develop standardized guidelines for managing R/R-P HR-NB. We also will develop Spanish-specific guidelines to further optimize treatment strategies in our setting. We highlight networking in our country, as half of the events were discussed and/or treated in six of the highest recruiting centres, with the aim of improving the quality of care and providing equal access to the best and most innovative therapies. Additional measures to further improve management and ensure these patients benefit from the collective experience are being implemented, such as quick consultations through the SEHOP web platform.

Salvage regimens commonly used in our cohort involved the administration of temozolomide, irinotecan or topotecan, similarly to recent CTs, which have also aimed to enhance outcomes by incorporating bevacizumab or anti-GD2 antibody [26, 27]. In addition, the use of chemotherapy in combination with anti-GD2 antibodies has shown high effectiveness, particularly in patients with refractory disease [26, 28, 29]. In our population, the use of anti-GD2 antibodies was related with a higher survival: OS 48.4% vs. 19.4% (*P* = 0.012) and EFS 35.5% vs. 8.3% (*P* = *0.007*). Collaborative groups are currently investigating these strategies in larger trials to determine their definitive role in this setting (NCT05272371, NCT03794349, NCT04560166, NCT06013618, NCT04211675).

Similar to front-line treatment approaches, a consolidation strategy may prove to be beneficial for R/R-P patients [30]. In our cohort, patients who underwent ASCR exhibited better outcomes, which could be explained by the fact that the performance of ASCR was associated to refractory and first relapse/progression events. However, these results may be subject to bias, as only responders in good clinical condition were eligible. With respect to local treatment, patients who underwent surgery + RT had better OS although again, there could be a selection bias.

Although we found a high rate of long-term survival in responders (median not reached for CR and PR, and of 20 months for SD), two-thirds of the R/R-P episodes were unable to achieve even SD with salvage therapies, highlighting the need for new treatments in this population.

Less than half of the episodes of R/R-P disease from our study were enrolled in CTs. Data regarding the reasons for low participation of neuroblastoma patients in trials are scarce. Broadening inclusion criteria or implementing measures to motivate individual physicians have been suggested to improve recruitment rates [23]. Molecular tumor boards (MTBs) have been implemented in Spain in 2024, aiming to discuss genomic findings and available clinical trials for each patient.

This study has important limitations. First, the small number of patients and the considerable heterogeneity hinders drawing conclusions on all factors influencing outcomes although we believe our sample to be representative since it is near the theoretical national annual incidence. Second, the retrospective nature of data collection introduces the possibility of missing information. In addition, the short follow-up due to the attempt of reflecting the outcome of the most recent R/R-P treatment strategies require caution when interpreting results.

In conclusion, our data show how R/R-P HR-NB treatment still has a high failure rate, although certain strategies, such as immunotherapy and intensive multimodal treatment at refractory or relapse/progression, can achieve prolonged survival in responders. Our results aim chemoimmunotherapy as the best candidate for treatment in this setting, followed by a consolidation and local treatment. Refractory patients, and those with late relapses, may experience better outcomes. There is urgent need for better implement molecular profiling and CT accrual in the R/R-P HR-NB in our country which could be fostered with MTB, patient advocacy engagement and development of national and international guidelines to ultimately improve outcomes for this population.

## Supplementary Information

Below is the link to the electronic supplementary material.

Supplementary file1 (PDF 448 KB)

Supplementary file2 (PDF 180 KB)

## Data Availability

Information used to develop this study and publication required to reanalyze the data reported in this paper is available from the lead contact upon request.
