# Correction: Management and outcome of children with high‑risk neuroblastoma: insights from the Spanish Society of Pediatric Hematology and Oncology (SEHOP) neuroblastoma group on refractory and relapse/progressive disease

**DOI:** 10.1007/s12094-025-03912-2

**Published:** 2025-04-09

**Authors:** Blanca Martínez de las Heras, Pedro M Rubio-Aparicio, Alba Rubio-San-Simón, Lucas Moreno, Paula Mazorra, Ricardo López Almaraz, Mercedes Llempén López, Julia Balaguer Guill, Vanessa Segura, Mar Bermúdez, Irene Jiménez, Désirée Ramal, Adela Cañete

**Affiliations:** 1 Pediatric Hemato-Oncology Department, Hospital Universitario y Politécnico La Fe, European Reference Network PAEDCAN member, Valencia, Spain; 2 Pediatric Hemato-Oncology Department, Hospital Universitario La Paz, European Reference Network PAEDCAN member, Madrid, Spain; 3 Pediatric Hemato-Oncology Department, Hospital Infantil Universitario Niño Jesús, Madrid, Spain; 4 Pediatric Department, Hospital Universitario Vall d´Hebron, European Reference Network PAEDCAN member, Barcelona, Spain; 5 Pediatric Onco-Hematology Unit, Hospital Cruces, and Pediatric Oncology Group Biobizkaia Health Research Institute, Barakaldo, Spain; 6 Pediatric Oncology Department, Hospital Universitario Virgen del Rocío, European Reference Network PAEDCAN member, Sevilla, Spain; 7 Clinical and Translational Oncology Research Group, Investigation Institute La Fe, Valencia, Spain; 8 Pediatric Hematology-Oncology Department, Hospital Clínico Universitario Virgen de la Arrixaca, Murcia, Spain

**Correction: Clinical and Translational Oncology** 10.1007/s12094-025-03853-w

In this article the statement in the Funding information section was incorrectly given as ‘Publication funded by the European Union-Next GenerationEU’ and should have read ‘This study has been co-funded by Instituto de Salud Carlos III (ISCIII) through the project “ICI21/00098” with funds from Plan de Recuperación, Transformación y Resiliencia and “Funded by the European Union – NextGenerationEU”’.

The original article has been corrected.

