# Supplementary material for: Management and outcome of children with high-risk neuroblastoma: insights from the Spanish Society of Pediatric Hematology and Oncology (SEHOP) neuroblastoma group on refractory and relapse/progressive disease
Source: Clin Transl Oncol. 2025 Feb 25;27(8):3421–31. doi: 10.1007/s12094-025-03853-w (PMC12259807; doi:10.1007/s12094-025-03853-w)
Supplement: Supplementary file 1 — Supplementary file1 (PDF 448 KB) [file 12094_2025_3853_MOESM1_ESM.pdf]

Kaplan-Meier OS ALL CASES

Avisos

La solicitud TEST no es válida porque no se ha especificado una variable de factor.
La solicitud COMPARISON no es válida porque no se ha especificado una variable de factor.

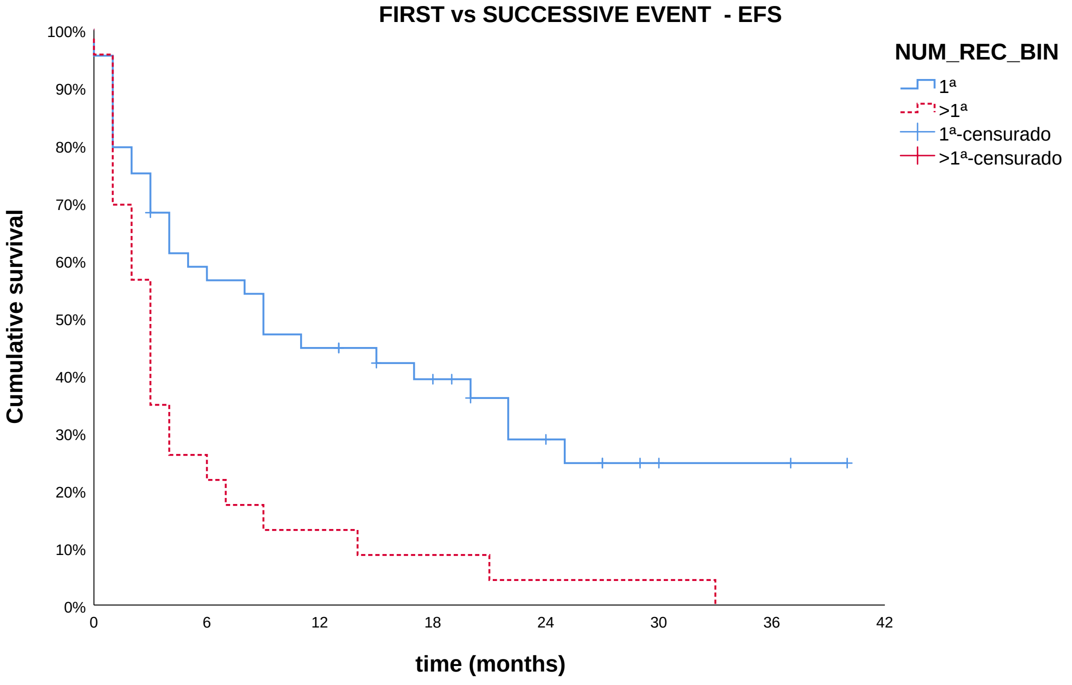

Resumen de procesamiento de casos

N total	N de eventos	Censurado	
		N	Porcentaje
67	45	22	32,8%

Medias y medianas para el tiempo de SUPERVIVENCIA GLOBAL

Media[a]				Mediana	
		Intervalo de confianza de 95 %			
Estimación	Desv. Error	Límite inferior	Límite superior	Estimación	Desv. Error
17,715	2,170	13,461	21,969	9,000	1,327

Medias y medianas para el tiempo de SUPERVIVENCIA GLOBAL

Mediana	
Intervalo de confianza de 95 %	
Límite inferior	Límite superior
6,399	11,601

a. La estimación está limitada al tiempo de supervivencia más largo, si está censurado.

Tabla de supervivencia

	Hora	Estado	Proporción acumulada que sobrevive en el tiempo		N de eventos acumulados	N de casos restantes
			Estimación	Desv. Error		
1	,000	NO	.	.	1	66
2	,000	NO	.	.	2	65
3	,000	NO	,955	,025	3	64
4	1,000	NO	.	.	4	63
5	1,000	NO	.	.	5	62
6	1,000	NO	.	.	6	61
7	1,000	NO	.	.	7	60
8	1,000	NO	,881	,040	8	59
9	2,000	NO	.	.	9	58
10	2,000	NO	.	.	10	57
11	2,000	NO	,836	,045	11	56
12	3,000	NO	.	.	12	55
13	3,000	NO	.	.	13	54
14	3,000	NO	.	.	14	53
15	3,000	NO	,776	,051	15	52
16	3,000	YES	.	.	15	51
17	4,000	NO	.	.	16	50
18	4,000	NO	.	.	17	49
19	4,000	NO	.	.	18	48
20	4,000	NO	,715	,055	19	47
21	4,000	YES	.	.	19	46
22	5,000	NO	.	.	20	45
23	5,000	NO	,684	,057	21	44
24	6,000	NO	.	.	22	43
25	6,000	NO	.	.	23	42
26	6,000	NO	,638	,059	24	41
27	7,000	NO	,622	,060	25	40

Tabla de supervivencia

	Hora	Estado	Proporción acumulada que sobrevive en el tiempo		N de eventos acumulados	N de casos restantes
			Estimación	Desv. Error		
28	8,000	NO	.	.	26	39
29	8,000	NO	.	.	27	38
30	8,000	NO	.	.	28	37
31	8,000	NO	.	.	29	36
32	8,000	NO	.	.	30	35
33	8,000	NO	,529	,062	31	34
34	9,000	NO	.	.	32	33
35	9,000	NO	.	.	33	32
36	9,000	NO	,482	,062	34	31
37	10,000	NO	.	.	35	30
38	10,000	NO	,451	,062	36	29
39	11,000	NO	,435	,061	37	28
40	12,000	NO	,420	,061	38	27
41	13,000	NO	,404	,061	39	26
42	13,000	YES	.	.	39	25
43	13,000	YES	.	.	39	24
44	15,000	YES	.	.	39	23
45	15,000	YES	.	.	39	22
46	18,000	YES	.	.	39	21
47	18,000	YES	.	.	39	20
48	19,000	YES	.	.	39	19
49	20,000	NO	.	.	40	18
50	20,000	NO	,362	,061	41	17
51	20,000	YES	.	.	41	16
52	21,000	NO	.	.	42	15
53	21,000	NO	,317	,062	43	14
54	23,000	YES	.	.	43	13
55	24,000	YES	.	.	43	12
56	24,000	YES	.	.	43	11
57	25,000	NO	,288	,062	44	10
58	27,000	YES	.	.	44	9
59	27,000	YES	.	.	44	8
60	28,000	YES	.	.	44	7
61	29,000	YES	.	.	44	6
62	30,000	YES	.	.	44	5
63	33,000	NO	,230	,072	45	4

Tabla de supervivencia

	Hora	Estado	Proporción acumulada que sobrevive en el tiempo		N de eventos acumulados	N de casos restantes
			Estimación	Desv. Error		
64	36,000	YES	.	.	45	3
65	37,000	YES	.	.	45	2
66	40,000	YES	.	.	45	1
67	44,000	YES	.	.	45	0

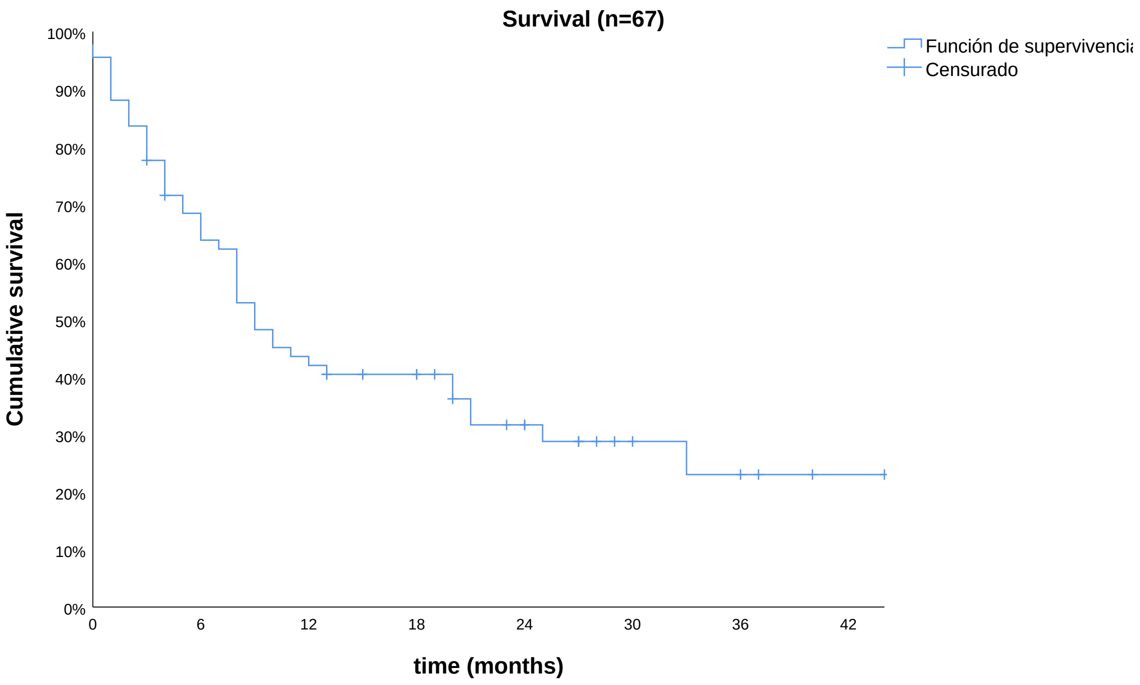

Análisis de supervivencia

Variable de supervivencia : Meses de entrada en estudio a última vis ita/exitus

Tabla de mortalidad[a]

Hora de inicio del intervalo	Número que entra en el intervalo	Número de retirada durante el intervalo	Número expuesto a riesgo	Número de eventos terminales
0	67	2	66,000	21
6	44	0	44,000	16
12	28	4	26,000	2
18	22	5	19,500	4
24	13	6	10,000	1
30	6	1	5,500	1
36	4	3	2,500	0
42	1	1	,500	0

Tabla de mortalidad[a]

Hora de inicio del intervalo	Proporción que termina	Proporción que sobrevive	Proporción acumulada que sobrevive al final del intervalo	Error estándar de la proporción acumulada que perdura al final del intervalo
0	,32	,68	,68	,06
6	,36	,64	,43	,06
12	,08	,92	,40	,06
18	,21	,79	,32	,06
24	,10	,90	,29	,06
30	,18	,82	,23	,07
36	,00	1,00	,23	,07
42	,00	1,00	,23	,07

Tabla de mortalidad[a]

Hora de inicio del intervalo	Densidad de probabilidad	Error estándar de la densidad de probabilidad	Índice de riesgo	Error estándar del índice de riesgo
0	,053	,010	,06	,01
6	,041	,009	,07	,02
12	,006	,004	,01	,01
18	,014	,006	,04	,02
24	,005	,005	,02	,02
30	,009	,008	,03	,03
36	,000	,000	,00	,00
42	,000	,000	,00	,00

a. La mediana del tiempo de supervivencia es 10,4000

Kaplan-Meier EFS ALL CASES

Resumen de procesamiento de casos

N total	N de eventos	Censurado	
		N	Porcentaje
67	53	14	20,9%

Tabla de supervivencia

	Hora	Estado	Proporción acumulada que sobrevive en el tiempo		N de eventos acumulados	N de casos restantes
			Estimación	Desv. Error		
1	,000	NO	.	.	1	66
2	,000	NO	.	.	2	65
3	,000	NO	,955	,025	3	64
4	1,000	NO	.	.	4	63
5	1,000	NO	.	.	5	62
6	1,000	NO	.	.	6	61
7	1,000	NO	.	.	7	60
8	1,000	NO	.	.	8	59
9	1,000	NO	.	.	9	58
10	1,000	NO	.	.	10	57
11	1,000	NO	.	.	11	56
12	1,000	NO	.	.	12	55
13	1,000	NO	.	.	13	54
14	1,000	NO	.	.	14	53
15	1,000	NO	.	.	15	52
16	1,000	NO	,761	,052	16	51
17	2,000	NO	.	.	17	50
18	2,000	NO	.	.	18	49
19	2,000	NO	.	.	19	48
20	2,000	NO	.	.	20	47
21	2,000	NO	,687	,057	21	46
22	3,000	NO	.	.	22	45
23	3,000	NO	.	.	23	44
24	3,000	NO	.	.	24	43
25	3,000	NO	.	.	25	42
26	3,000	NO	.	.	26	41
27	3,000	NO	.	.	27	40
28	3,000	NO	.	.	28	39
29	3,000	NO	,567	,061	29	38
30	3,000	YES	.	.	29	37
31	4,000	NO	.	.	30	36
32	4,000	NO	.	.	31	35
33	4,000	NO	.	.	32	34
34	4,000	NO	.	.	33	33
35	4,000	NO	,491	,061	34	32
36	5,000	NO	,475	,061	35	31

Tabla de supervivencia

	Hora	Estado	Proporción acumulada que sobrevive en el tiempo		N de eventos acumulados	N de casos restantes
			Estimación	Desv. Error		
37	6,000	NO	.	.	36	30
38	6,000	NO	,445	,061	37	29
39	7,000	NO	,429	,061	38	28
40	8,000	NO	,414	,061	39	27
41	9,000	NO	.	.	40	26
42	9,000	NO	.	.	41	25
43	9,000	NO	.	.	42	24
44	9,000	NO	,353	,059	43	23
45	11,000	NO	,337	,058	44	22
46	13,000	YES	.	.	44	21
47	13,000	YES	.	.	44	20
48	14,000	NO	,320	,058	45	19
49	15,000	NO	,304	,057	46	18
50	15,000	YES	.	.	46	17
51	17,000	NO	,286	,056	47	16
52	18,000	YES	.	.	47	15
53	19,000	YES	.	.	47	14
54	20,000	NO	,265	,056	48	13
55	20,000	YES	.	.	48	12
56	21,000	NO	,243	,056	49	11
57	22,000	NO	.	.	50	10
58	22,000	NO	,199	,054	51	9
59	24,000	YES	.	.	51	8
60	25,000	NO	,174	,052	52	7
61	27,000	YES	.	.	52	6
62	27,000	YES	.	.	52	5
63	29,000	YES	.	.	52	4
64	30,000	YES	.	.	52	3
65	33,000	NO	,116	,059	53	2
66	37,000	YES	.	.	53	1
67	40,000	YES	.	.	53	0

Medias y medianas para el tiempo de supervivencia

Media[a]				Mediana	
		Intervalo de confianza de 95 %			
Estimación	Desv. Error	Límite inferior	Límite superior	Estimación	Desv. Error
12,035	1,734	8,636	15,434	4,000	1,499

Medias y medianas para el tiempo de supervivencia

Mediana	
Intervalo de confianza de 95 %	
Límite inferior	Límite superior
1,061	6,939

a. La estimación está limitada al tiempo de supervivencia más largo, si está censurado.

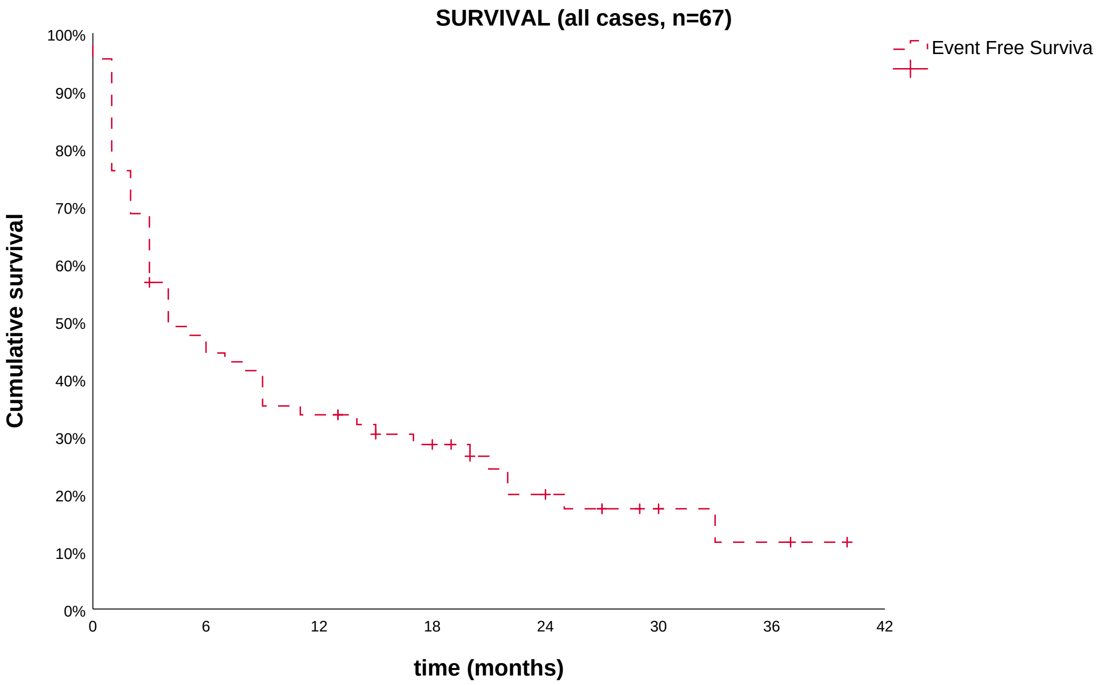

Análisis de supervivencia

Variable de supervivencia : SLE (meses, episodios)

Tabla de mortalidad[a]

Hora de inicio del intervalo	Número que entra en el intervalo	Número de retirada durante el intervalo	Número expuesto a riesgo	Número de eventos terminales
0	64	1	63,500	35
6	28	0	28,000	9
12	19	3	17,500	3
18	13	3	11,500	2
24	8	4	6,000	0
30	4	1	3,500	1
36	2	2	1,000	0

Tabla de mortalidad[a]

Hora de inicio del intervalo	Proporción que termina	Proporción que sobrevive	Proporción acumulada que sobrevive al final del intervalo	Error estándar de la proporción acumulada que perdura al final del intervalo
0	,55	,45	,45	,06
6	,32	,68	,30	,06
12	,17	,83	,25	,06
18	,17	,83	,21	,05
24	,00	1,00	,21	,05
30	,29	,71	,15	,06
36	,00	1,00	,15	,06

Tabla de mortalidad[a]

Hora de inicio del intervalo	Densidad de probabilidad	Error estándar de la densidad de probabilidad	Índice de riesgo	Error estándar del índice de riesgo
0	,092	,010	,13	,02
6	,024	,007	,06	,02
12	,009	,005	,03	,02
18	,007	,005	,03	,02
24	,000	,000	,00	,00
30	,010	,009	,06	,05
36	,000	,000	,00	,00

a. La mediana del tiempo de supervivencia es 5,4429

Kaplan-Meier OS PD VS REF

Resumen de procesamiento de casos

REC/REF	N total	N de eventos	Censurado N	Censurado Porcentaje
PD	60	43	17	28,3%
REFRACTORY	7	2	5	71,4%
Global	67	45	22	32,8%

Medias y medianas para el tiempo de supervivencia

REC/REF	Media[a] Estimación	Media[a] Desv. Error	Media[a] Intervalo de confianza de 95 % Límite inferior	Media[a] Intervalo de confianza de 95 % Límite superior	Mediana Estimación
PD	16,037	2,205	11,714	20,359	8,000
REFRACTORY	30,667	5,217	20,440	40,893	.
Global	17,715	2,170	13,461	21,969	9,000

Medias y medianas para el tiempo de supervivencia

REC/REF	Mediana Desv. Error	Mediana Intervalo de confianza de 95 % Límite inferior	Mediana Intervalo de confianza de 95 % Límite superior
PD	,953	6,132	9,868
REFRACTORY	.	.	.
Global	1,327	6,399	11,601

a. La estimación está limitada al tiempo de supervivencia más largo, si está censurado.

Comparaciones globales

	Chi-cuadrado	gl	Sig.
Log Rank (Mantel-Cox)	4,222	1	,040

Prueba de igualdad de distribuciones de supervivencia para los distintos niveles de REC/REF.

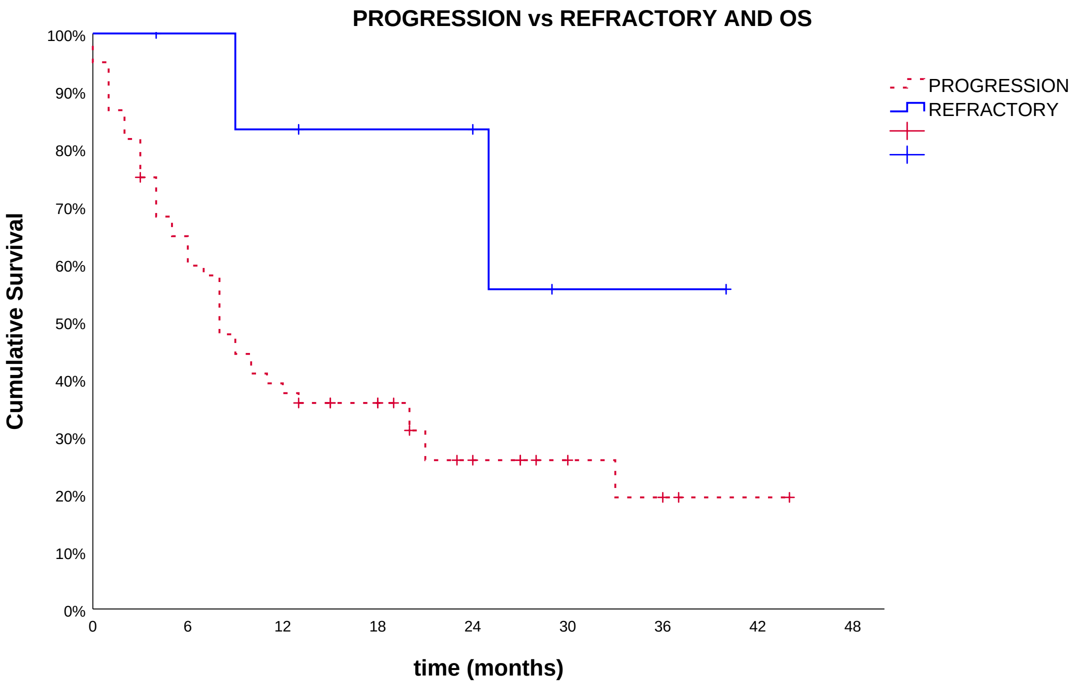

Análisis de supervivencia

Variable de supervivencia : Meses de entrada en estudio a última vis ita/exitus

Tabla de mortalidad

Controles de primer orden		Hora de inicio del intervalo	Número que entra en el intervalo	Número de retirada durante el intervalo
REC/REF	PD	0	60	1
		6	38	0
		12	23	3
		18	18	5
		24	9	4
		30	5	1
		36	3	2
		42	1	1
	REFRACTORY	0	7	1
		6	6	0
		12	5	1
		18	4	0
		24	4	2
		30	1	0
		36	1	1

Tabla de mortalidad

Controles de primer orden		Hora de inicio del intervalo	Número expuesto a riesgo	Número de eventos terminales
REC/REF	PD	0	59,500	21
		6	38,000	15
		12	21,500	2
		18	15,500	4
		24	7,000	0
		30	4,500	1
		36	2,000	0
		42	,500	0
	REFRACTORY	0	6,500	0
		6	6,000	1
		12	4,500	0
		18	4,000	0
		24	3,000	1
		30	1,000	0
		36	,500	0

Tabla de mortalidad

Controles de primer orden		Hora de inicio del intervalo	Proporción que termina	Proporción que sobrevive
REC/REF	PD	0	,35	,65
		6	,39	,61
		12	,09	,91
		18	,26	,74
		24	,00	1,00
		30	,22	,78
		36	,00	1,00
		42	,00	1,00
	REFRACTORY	0	,00	1,00
		6	,17	,83
		12	,00	1,00
		18	,00	1,00
		24	,33	,67
		30	,00	1,00
		36	,00	1,00

Tabla de mortalidad

Controles de primer orden		Hora de inicio del intervalo	Proporción acumulada que sobrevive al final del intervalo	Error estándar de la proporción acumulada que perdura al final del intervalo
REC/REF	PD	0	,65	,06
		6	,39	,06
		12	,36	,06
		18	,26	,06
		24	,26	,06
		30	,20	,07
		36	,20	,07
		42	,20	,07
	REFRACTORY	0	1,00	,00
		6	,83	,15
		12	,83	,15
		18	,83	,15
		24	,56	,25
		30	,56	,25
		36	,56	,25

Tabla de mortalidad

Controles de primer orden		Hora de inicio del intervalo	Densidad de probabilidad	Error estándar de la densidad de probabilidad
REC/REF	PD	0	,059	,010
		6	,043	,009
		12	,006	,004
		18	,015	,007
		24	,000	,000
		30	,010	,009
		36	,000	,000
		42	,000	,000
	REFRACTORY	0	,000	,000
		6	,028	,025
		12	,000	,000
		18	,000	,000
		24	,046	,039
		30	,000	,000
		36	,000	,000

Tabla de mortalidad

Controles de primer orden		Hora de inicio del intervalo	Índice de riesgo	Error estándar del índice de riesgo
REC/REF	PD	0	,07	,02
		6	,08	,02
		12	,02	,01
		18	,05	,02
		24	,00	,00
		30	,04	,04
		36	,00	,00
		42	,00	,00
	REFRACTORY	0	,00	,00
		6	,03	,03
		12	,00	,00
		18	,00	,00
		24	,07	,07
		30	,00	,00
		36	,00	,00

Kaplan-Meier EFS PD VS REF

Resumen de procesamiento de casos

REC/REF	N total	N de eventos	Censurado	
			N	Porcentaje
PD	60	50	10	16,7%
REFRACTORY	7	3	4	57,1%
Global	67	53	14	20,9%

Medias y medianas para el tiempo de supervivencia

	Media[a]				Mediana
			Intervalo de confianza de 95 %		
REC/REF	Estimación	Desv. Error	Límite inferior	Límite superior	Estimación
PD	10,074	1,563	7,010	13,138	4,000
REFRACTORY	26,143	6,043	14,298	37,988	25,000
Global	12,035	1,734	8,636	15,434	4,000

Medias y medianas para el tiempo de supervivencia

	Mediana		
		Intervalo de confianza de 95 %	
REC/REF	Desv. Error	Límite inferior	Límite superior
PD	,958	2,122	5,878
REFRACTORY	.	.	.
Global	1,499	1,061	6,939

a. La estimación está limitada al tiempo de supervivencia más largo, si está censurado.

Comparaciones globales

	Chi-cuadrado	gl	Sig.
Log Rank (Mantel-Cox)	5,599	1	,018

Prueba de igualdad de distribuciones de supervivencia para los distintos niveles de REC/REF.

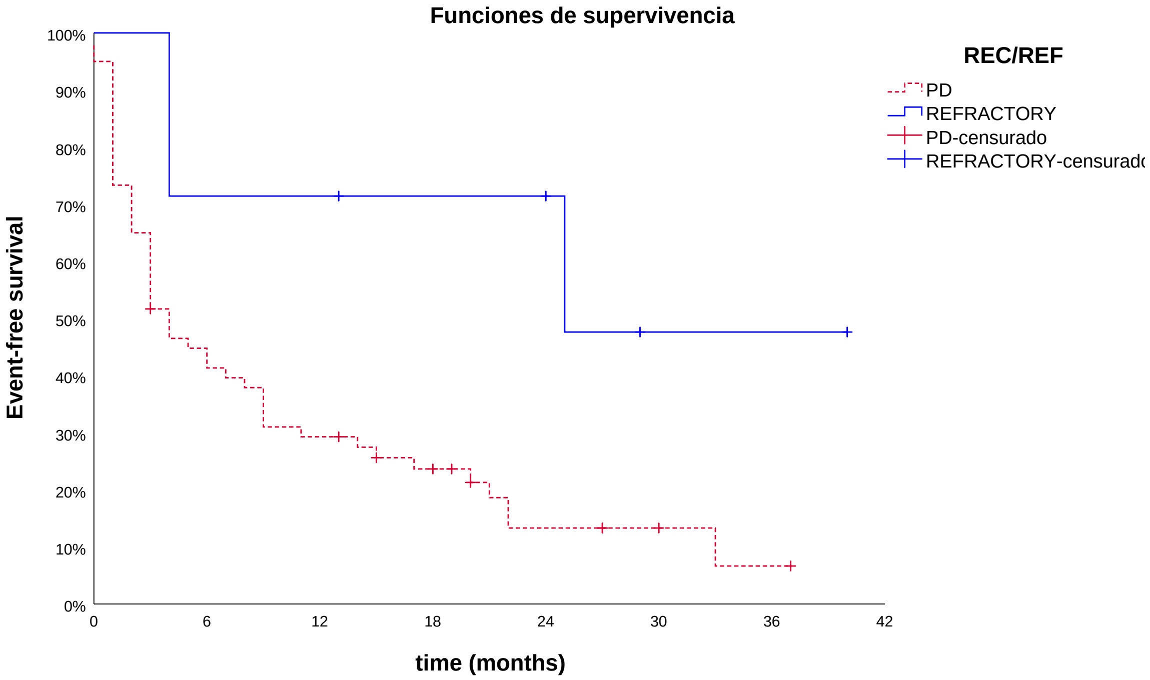

Análisis de supervivencia

Variable de supervivencia : SLE (meses, episodios)

Tabla de mortalidad

Controles de primer orden		Hora de inicio del intervalo	Número que entra en el intervalo	Número de retirada durante el intervalo
REC/REF	PD	0	58	1
		6	24	0
		12	15	2
		18	10	3
		24	5	2
		30	3	1
		36	1	1
	REFRACTORY	0	6	0
		6	4	0
		12	4	1
		18	3	0
		24	3	2
		30	1	0
		36	1	1

Tabla de mortalidad

Controles de primer orden		Hora de inicio del intervalo	Número expuesto a riesgo	Número de eventos terminales
REC/REF	PD	0	57,500	33
		6	24,000	9
		12	14,000	3
		18	8,500	2
		24	4,000	0
		30	2,500	1
		36	,500	0
	REFRACTORY	0	6,000	2
		6	4,000	0
		12	3,500	0
		18	3,000	0
		24	2,000	0
		30	1,000	0
		36	,500	0

Tabla de mortalidad

Controles de primer orden		Hora de inicio del intervalo	Proporción que termina	Proporción que sobrevive
REC/REF	PD	0	,57	,43
		6	,38	,63
		12	,21	,79
		18	,24	,76
		24	,00	1,00
		30	,40	,60
		36	,00	1,00
	REFRACTORY	0	,33	,67
		6	,00	1,00
		12	,00	1,00
		18	,00	1,00
		24	,00	1,00
		30	,00	1,00
		36	,00	1,00

Tabla de mortalidad

Controles de primer orden		Hora de inicio del intervalo	Proporción acumulada que sobrevive al final del intervalo	Error estándar de la proporción acumulada que perdura al final del intervalo
REC/REF	PD	0	,43	,07
		6	,27	,06
		12	,21	,05
		18	,16	,05
		24	,16	,05
		30	,10	,06
		36	,10	,06
	REFRACTORY	0	,67	,19
		6	,67	,19
		12	,67	,19
		18	,67	,19
		24	,67	,19
		30	,67	,19
		36	,67	,19

Tabla de mortalidad

Controles de primer orden		Hora de inicio del intervalo	Densidad de probabilidad	Error estándar de la densidad de probabilidad
REC/REF	PD	0	,096	,011
		6	,027	,008
		12	,010	,005
		18	,008	,006
		24	,000	,000
		30	,011	,009
		36	,000	,000
	REFRACTORY	0	,056	,032
		6	,000	,000
		12	,000	,000
		18	,000	,000
		24	,000	,000
		30	,000	,000
		36	,000	,000

Tabla de mortalidad

Controles de primer orden		Hora de inicio del intervalo	Índice de riesgo	Error estándar del índice de riesgo
REC/REF	PD	0	,13	,02
		6	,08	,02
		12	,04	,02
		18	,04	,03
		24	,00	,00
		30	,08	,08
		36	,00	,00
	REFRACTORY	0	,07	,05
		6	,00	,00
		12	,00	,00
		18	,00	,00
		24	,00	,00
		30	,00	,00
		36	,00	,00

Kaplan-Meier OS 1ST VS >1ST

Resumen de procesamiento de casos

NUM_REC_BIN	N total	N de eventos	Censurado	
			N	Porcentaje
1ª	44	24	20	45,5%
>1ª	23	21	2	8,7%
Global	67	45	22	32,8%

Medias y medianas para el tiempo de supervivencia

NUM_REC_BIN	Media[a]				Mediana
			Intervalo de confianza de 95 %		
	Estimación	Desv. Error	Límite inferior	Límite superior	Estimación
1ª	22,342	2,827	16,801	27,883	20,000
>1ª	8,304	2,015	4,354	12,255	5,000
Global	17,715	2,170	13,461	21,969	9,000

Medias y medianas para el tiempo de supervivencia

NUM_REC_BIN	Mediana		
		Intervalo de confianza de 95 %	
	Desv. Error	Límite inferior	Límite superior
1ª	6,101	8,043	31,957
>1ª	,951	3,136	6,864
Global	1,327	6,399	11,601

a. La estimación está limitada al tiempo de supervivencia más largo, si está censurado.

Comparaciones globales

	Chi-cuadrado	gl	Sig.
Log Rank (Mantel-Cox)	11,897	1	,001

Prueba de igualdad de distribuciones de supervivencia para los distintos niveles de NUM_REC_BIN.

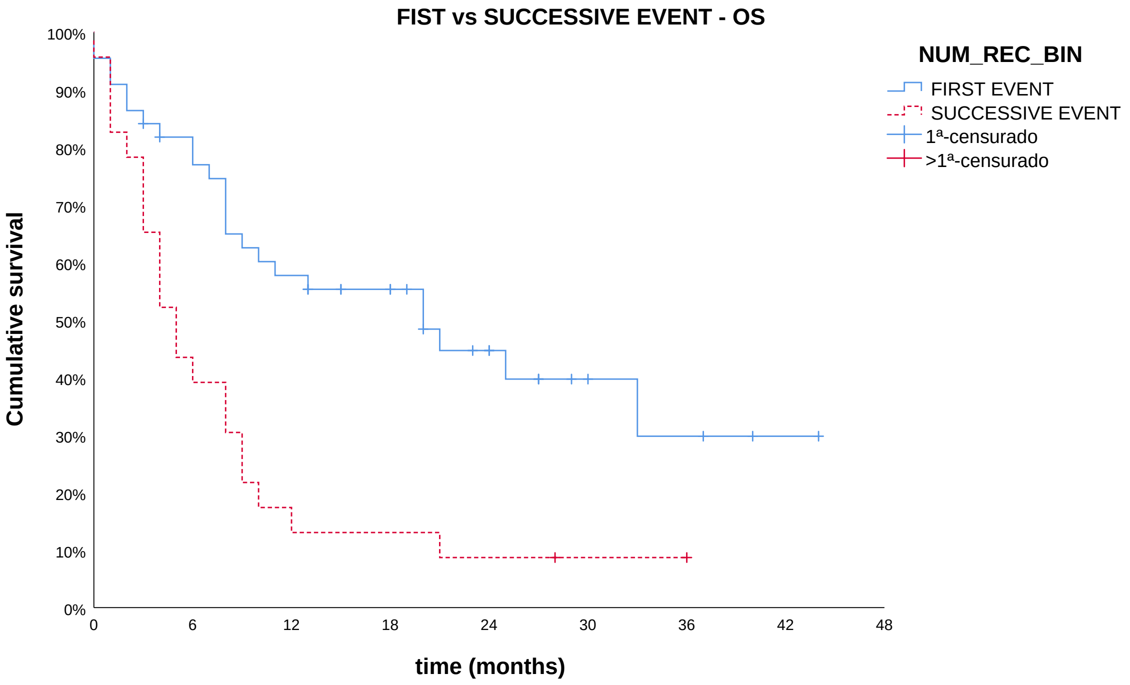

Análisis de supervivencia

Variable de supervivencia : Meses de entrada en estudio a última vis ita/exitus

Tabla de mortalidad

Controles de primer orden		Hora de inicio del intervalo	Número que entra en el intervalo	Número de retirada durante el intervalo
NUM_REC_BIN	1ª	0	44	2
		6	34	0
		12	24	4
		18	19	5
		24	11	5
		30	5	1
		36	3	2
		42	1	1
	>1ª	0	23	0
		6	10	0
		12	4	0
		18	3	0
		24	2	1
		30	1	0
		36	1	1

Tabla de mortalidad

Controles de primer orden		Hora de inicio del intervalo	Número expuesto a riesgo	Número de eventos terminales
NUM_REC_BIN	1ª	0	43,000	8
		6	34,000	10
		12	22,000	1
		18	16,500	3
		24	8,500	1
		30	4,500	1
		36	2,000	0
		42	,500	0
	>1ª	0	23,000	13
		6	10,000	6
		12	4,000	1
		18	3,000	1
		24	1,500	0
		30	1,000	0
		36	,500	0

Tabla de mortalidad

Controles de primer orden		Hora de inicio del intervalo	Proporción que termina	Proporción que sobrevive
NUM_REC_BIN	1ª	0	,19	,81
		6	,29	,71
		12	,05	,95
		18	,18	,82
		24	,12	,88
		30	,22	,78
		36	,00	1,00
		42	,00	1,00
	>1ª	0	,57	,43
		6	,60	,40
		12	,25	,75
		18	,33	,67
		24	,00	1,00
		30	,00	1,00
		36	,00	1,00

Tabla de mortalidad

Controles de primer orden		Hora de inicio del intervalo	Proporción acumulada que sobrevive al final del intervalo	Error estándar de la proporción acumulada que perdura al final del intervalo
NUM_REC_BIN	1ª	0	,81	,06
		6	,57	,08
		12	,55	,08
		18	,45	,08
		24	,40	,09
		30	,31	,10
		36	,31	,10
		42	,31	,10
	>1ª	0	,43	,10
		6	,17	,08
		12	,13	,07
		18	,09	,06
		24	,09	,06
		30	,09	,06
		36	,09	,06

Tabla de mortalidad

Controles de primer orden		Hora de inicio del intervalo	Densidad de probabilidad	Error estándar de la densidad de probabilidad
NUM_REC_BIN	1ª	0	,031	,010
		6	,040	,011
		12	,004	,004
		18	,017	,009
		24	,009	,008
		30	,015	,013
		36	,000	,000
		42	,000	,000
	>1ª	0	,094	,017
		6	,043	,015
		12	,007	,007
		18	,007	,007
		24	,000	,000
		30	,000	,000
		36	,000	,000

Tabla de mortalidad

Controles de primer orden		Hora de inicio del intervalo	Índice de riesgo	Error estándar del índice de riesgo
NUM_REC_BIN	1ª	0	,03	,01
		6	,06	,02
		12	,01	,01
		18	,03	,02
		24	,02	,02
		30	,04	,04
		36	,00	,00
		42	,00	,00
	>1ª	0	,13	,03
		6	,14	,05
		12	,05	,05
		18	,07	,07
		24	,00	,00
		30	,00	,00
		36	,00	,00

Kaplan-Meier EFS 1ST VS >1ST

Resumen de procesamiento de casos

NUM_REC_BIN	N total	N de eventos	Censurado	
			N	Porcentaje
1ª	44	30	14	31,8%
>1ª	23	23	0	0,0%
Global	67	53	14	20,9%

Medias y medianas para el tiempo de supervivencia

	Media[a]				Mediana
			Intervalo de confianza de 95 %		
NUM_REC_BIN	Estimación	Desv. Error	Límite inferior	Límite superior	Estimación
1ª	16,078	2,436	11,304	20,852	9,000
>1ª	5,435	1,608	2,284	8,586	3,000
Global	12,035	1,734	8,636	15,434	4,000

Medias y medianas para el tiempo de supervivencia

	Mediana		
		Intervalo de confianza de 95 %	
NUM_REC_BIN	Desv. Error	Límite inferior	Límite superior
1ª	3,233	2,664	15,336
>1ª	,457	2,105	3,895
Global	1,499	1,061	6,939

a. La estimación está limitada al tiempo de supervivencia más largo, si está censurado.

Comparaciones globales

	Chi-cuadrado	gl	Sig.
Log Rank (Mantel-Cox)	10,981	1	,001

Prueba de igualdad de distribuciones de supervivencia para los distintos niveles de NUM_REC_BIN.

Análisis de supervivencia

Variable de supervivencia : SLE (meses, episodios)

Tabla de mortalidad

Controles de primer orden		Hora de inicio del intervalo	Número que entra en el intervalo	Número de retirada durante el intervalo
NUM_REC_BIN	1ª	0	41	1
		6	22	0
		12	16	3
		18	11	3
		24	7	4
		30	3	1
		36	2	2
	>1ª	0	23	0
		6	6	0
		12	3	0
		18	2	0
		24	1	0
		30	1	0

Tabla de mortalidad

Controles de primer orden		Hora de inicio del intervalo	Número expuesto a riesgo	Número de eventos terminales
NUM_REC_BIN	1ª	0	40,500	18
		6	22,000	6
		12	14,500	2
		18	9,500	1
		24	5,000	0
		30	2,500	0
		36	1,000	0
	>1ª	0	23,000	17
		6	6,000	3
		12	3,000	1
		18	2,000	1
		24	1,000	0
		30	1,000	1

Tabla de mortalidad

Controles de primer orden		Hora de inicio del intervalo	Proporción que termina	Proporción que sobrevive
NUM_REC_BIN	1ª	0	,44	,56
		6	,27	,73
		12	,14	,86
		18	,11	,89
		24	,00	1,00
		30	,00	1,00
		36	,00	1,00
	>1ª	0	,74	,26
		6	,50	,50
		12	,33	,67
		18	,50	,50
		24	,00	1,00
		30	1,00	,00

Tabla de mortalidad

Controles de primer orden		Hora de inicio del intervalo	Proporción acumulada que sobrevive al final del intervalo	Error estándar de la proporción acumulada que perdura al final del intervalo
NUM_REC_BIN	1ª	0	,56	,08
		6	,40	,08
		12	,35	,08
		18	,31	,08
		24	,31	,08
		30	,31	,08
		36	,31	,08
	>1ª	0	,26	,09
		6	,13	,07
		12	,09	,06
		18	,04	,04
		24	,04	,04
		30	,00	,00

Tabla de mortalidad

Controles de primer orden		Hora de inicio del intervalo	Densidad de probabilidad	Error estándar de la densidad de probabilidad
NUM_REC_BIN	1ª	0	,074	,013
		6	,025	,009
		12	,009	,006
		18	,006	,006
		24	,000	,000
		30	,000	,000
		36	,000	,000
	>1ª	0	,123	,015
		6	,022	,012
		12	,007	,007
		18	,007	,007
		24	,000	,000
		30	,007	,007

Tabla de mortalidad

Controles de primer orden		Hora de inicio del intervalo	Índice de riesgo	Error estándar del índice de riesgo
NUM_REC_BIN	1ª	0	,10	,02
		6	,05	,02
		12	,02	,02
		18	,02	,02
		24	,00	,00
		30	,00	,00
		36	,00	,00
	>1ª	0	,20	,04
		6	,11	,06
		12	,07	,07
		18	,11	,10
		24	,00	,00
		30	,33	,00

Avisos

No se han calculado estadísticas porque todos los casos se han censurado.
No se han calculado estadísticas porque todos los casos se han censurado.

Resumen de procesamiento de casos

RESPUESTA	N total	N de eventos	Censurado N	Censurado Porcentaje
CR	12	0	12	100,0%
PR	4	0	4	100,0%
SD	7	4	3	42,9%
PD	43	41	2	4,7%
Global	66	45	21	31,8%

Comparaciones globales

	Chi-cuadrado	gl	Sig.
Log Rank (Mantel-Cox)	42,905	3	,000

Prueba de igualdad de distribuciones de supervivencia para los distintos niveles de RESPUESTA.

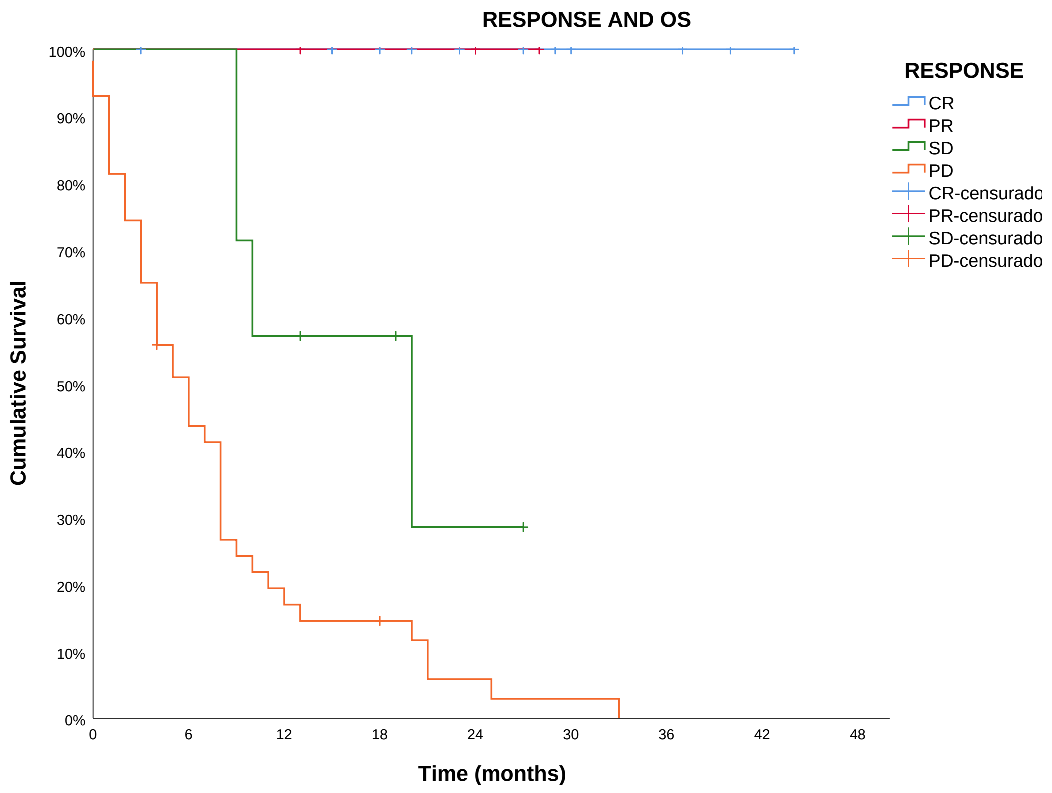

Análisis de supervivencia

Variable de supervivencia : Meses de entrada en estudio a última vis ita/exitus

Tabla de mortalidad

Controles de primer orden		Hora de inicio del intervalo	Número que entra en el intervalo	Número de retirada durante el intervalo
RESPUESTA	CR	0	12	1
		6	11	0
		12	11	2
		18	9	3
		24	6	2
		30	4	1
		36	3	2
		42	1	1
	PR	0	4	0
		6	4	0
		12	4	1
		18	3	0
		24	3	3
	SD	0	7	0
		6	7	0
		12	4	1
		18	3	1
		24	1	1
	PD	0	43	1
		6	21	0
		12	8	0
		18	6	1
		24	2	0
		30	1	0

Tabla de mortalidad

Controles de primer orden		Hora de inicio del intervalo	Número expuesto a riesgo	Número de eventos terminales
RESPUESTA	CR	0	11,500	0
		6	11,000	0
		12	10,000	0
		18	7,500	0
		24	5,000	0
		30	3,500	0
		36	2,000	0
		42	,500	0
	PR	0	4,000	0
		6	4,000	0
		12	3,500	0
		18	3,000	0
		24	1,500	0
	SD	0	7,000	0
		6	7,000	3
		12	3,500	0
		18	2,500	1
		24	,500	0
	PD	0	42,500	21
		6	21,000	13
		12	8,000	2
		18	5,500	3
		24	2,000	1
		30	1,000	1

Tabla de mortalidad

Controles de primer orden		Hora de inicio del intervalo	Proporción que termina	Proporción que sobrevive
RESPUESTA	CR	0	,00	1,00
		6	,00	1,00
		12	,00	1,00
		18	,00	1,00
		24	,00	1,00
		30	,00	1,00
		36	,00	1,00
		42	,00	1,00
	PR	0	,00	1,00
		6	,00	1,00
		12	,00	1,00
		18	,00	1,00
		24	,00	1,00
	SD	0	,00	1,00
		6	,43	,57
		12	,00	1,00
		18	,40	,60
		24	,00	1,00
	PD	0	,49	,51
		6	,62	,38
		12	,25	,75
		18	,55	,45
		24	,50	,50
		30	1,00	,00

Tabla de mortalidad

Controles de primer orden		Hora de inicio del intervalo	Proporción acumulada que sobrevive al final del intervalo	Error estándar de la proporción acumulada que perdura al final del intervalo
RESPUESTA	CR	0	1,00	,00
		6	1,00	,00
		12	1,00	,00
		18	1,00	,00
		24	1,00	,00
		30	1,00	,00
		36	1,00	,00
		42	1,00	,00
	PR	0	1,00	,00
		6	1,00	,00
		12	1,00	,00
		18	1,00	,00
		24	1,00	,00
	SD	0	1,00	,00
		6	,57	,19
		12	,57	,19
		18	,34	,21
		24	,34	,21
	PD	0	,51	,08
		6	,19	,06
		12	,14	,05
		18	,07	,04
		24	,03	,03
		30	,00	,00

Tabla de mortalidad

Controles de primer orden		Hora de inicio del intervalo	Densidad de probabilidad	Error estándar de la densidad de probabilidad
RESPUESTA	CR	0	,000	,000
		6	,000	,000
		12	,000	,000
		18	,000	,000
		24	,000	,000
		30	,000	,000
		36	,000	,000
		42	,000	,000
	PR	0	,000	,000
		6	,000	,000
		12	,000	,000
		18	,000	,000
		24	,000	,000
	SD	0	,000	,000
		6	,071	,031
		12	,000	,000
		18	,038	,032
		24	,000	,000
	PD	0	,082	,013
		6	,052	,012
		12	,008	,006
		18	,013	,007
		24	,005	,005
		30	,005	,005

Tabla de mortalidad

Controles de primer orden		Hora de inicio del intervalo	Índice de riesgo	Error estándar del índice de riesgo
RESPUESTA	CR	0	,00	,00
		6	,00	,00
		12	,00	,00
		18	,00	,00
		24	,00	,00
		30	,00	,00
		36	,00	,00
		42	,00	,00
	PR	0	,00	,00
		6	,00	,00
		12	,00	,00
		18	,00	,00
		24	,00	,00
	SD	0	,00	,00
		6	,09	,05
		12	,00	,00
		18	,08	,08
		24	,00	,00
	PD	0	,11	,02
		6	,15	,04
		12	,05	,03
		18	,13	,07
		24	,11	,10
		30	,33	,00

Kaplan-Meier EFS AND RESPONSE

Resumen de procesamiento de casos

RESPUESTA	N total	N de eventos	Censurado	
			N	Porcentaje
CR	12	3	9	75,0%
PR	4	2	2	50,0%
SD	7	4	3	42,9%
PD	43	43	0	0,0%
Global	66	52	14	21,2%

Medias y medianas para el tiempo de supervivencia

RESPUESTA	Media[a]				Mediana
	Estimación	Desv. Error	Intervalo de confianza de 95 %		Estimación
			Límite inferior	Límite superior	
CR	32,557	3,678	25,348	39,766	.
PR	22,333	,720	20,922	23,745	22,000
SD	15,571	3,779	8,165	22,978	9,000
PD	4,419	,885	2,684	6,153	3,000
Global	11,954	1,778	8,469	15,438	4,000

Medias y medianas para el tiempo de supervivencia

RESPUESTA	Mediana		
	Desv. Error	Intervalo de confianza de 95 %	
		Límite inferior	Límite superior
CR	.	.	.
PR	,816	20,400	23,600
SD	1,309	6,434	11,566
PD	,473	2,073	3,927
Global	1,487	1,086	6,914

a. La estimación está limitada al tiempo de supervivencia más largo, si está censurado.

Comparaciones globales

	Chi-cuadrado	gl	Sig.
Log Rank (Mantel-Cox)	40,955	3	,000

Prueba de igualdad de distribuciones de supervivencia para los distintos niveles de RESPUESTA.

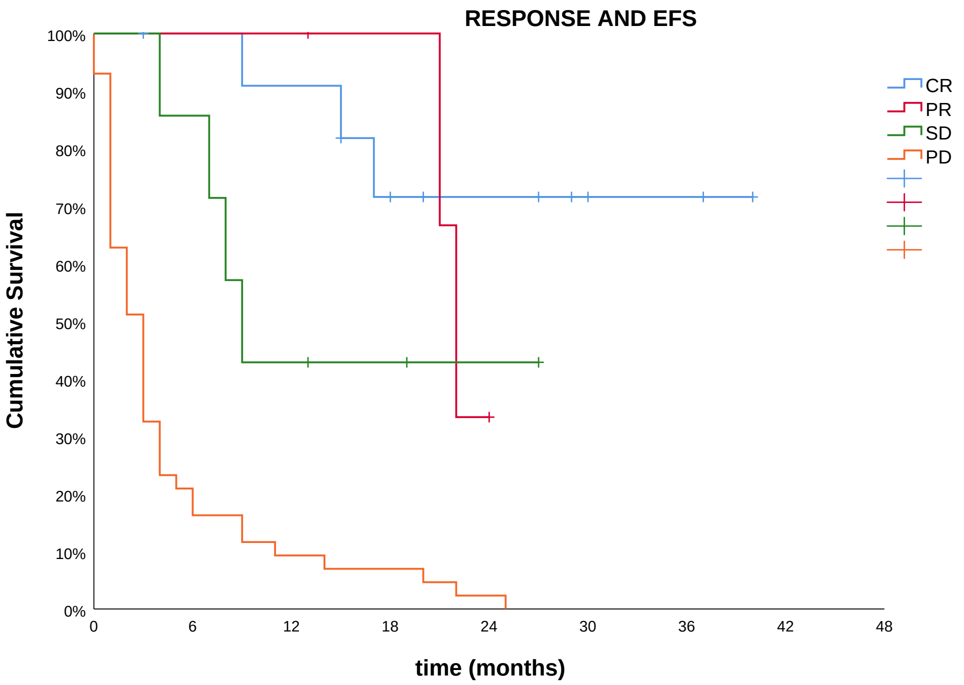

Análisis de supervivencia

Variable de supervivencia : SLE (meses, episodios)

Tabla de mortalidad

Controles de primer orden		Hora de inicio del intervalo	Número que entra en el intervalo	Número de retirada durante el intervalo
RESPUESTA	CR	0	12	1
		6	11	0
		12	10	1
		18	7	2
		24	5	2
		30	3	1
		36	2	2
	PR	0	4	0
		6	4	0
		12	4	1
		18	3	0
		24	1	1
	SD	0	7	0
		6	6	0

Tabla de mortalidad

Controles de primer orden		Hora de inicio del intervalo	Número expuesto a riesgo	Número de eventos terminales
RESPUESTA	CR	0	11,500	0
		6	11,000	1
		12	9,500	2
		18	6,000	0
		24	4,000	0
		30	2,500	0
		36	1,000	0
	PR	0	4,000	0
		6	4,000	0
		12	3,500	0
		18	3,000	2
		24	,500	0
	SD	0	7,000	1
		6	6,000	3

Tabla de mortalidad

Controles de primer orden		Hora de inicio del intervalo	Proporción que termina	Proporción que sobrevive
RESPUESTA	CR	0	,00	1,00
		6	,09	,91
		12	,21	,79
		18	,00	1,00
		24	,00	1,00
		30	,00	1,00
		36	,00	1,00
	PR	0	,00	1,00
		6	,00	1,00
		12	,00	1,00
		18	,67	,33
		24	,00	1,00
	SD	0	,14	,86
		6	,50	,50

Tabla de mortalidad

Controles de primer orden		Hora de inicio del intervalo	Proporción acumulada que sobrevive al final del intervalo	Error estándar de la proporción acumulada que perdura al final del intervalo
RESPUESTA	CR	0	1,00	,00
		6	,91	,09
		12	,72	,14
		18	,72	,14
		24	,72	,14
		30	,72	,14
		36	,72	,14
	PR	0	1,00	,00
		6	1,00	,00
		12	1,00	,00
		18	,33	,27
		24	,33	,27
	SD	0	,86	,13
		6	,43	,19

Tabla de mortalidad

Controles de primer orden		Hora de inicio del intervalo	Densidad de probabilidad	Error estándar de la densidad de probabilidad
RESPUESTA	CR	0	,000	,000
		6	,015	,014
		12	,032	,020
		18	,000	,000
		24	,000	,000
		30	,000	,000
		36	,000	,000
	PR	0	,000	,000
		6	,000	,000
		12	,000	,000
		18	,111	,045
		24	,000	,000
	SD	0	,024	,022
		6	,071	,031

Tabla de mortalidad

Controles de primer orden		Hora de inicio del intervalo	Índice de riesgo	Error estándar del índice de riesgo
RESPUESTA	CR	0	,00	,00
		6	,02	,02
		12	,04	,03
		18	,00	,00
		24	,00	,00
		30	,00	,00
		36	,00	,00
	PR	0	,00	,00
		6	,00	,00
		12	,00	,00
		18	,17	,10
		24	,00	,00
	SD	0	,03	,03
		6	,11	,06

Tabla de mortalidad

Controles de primer orden		Hora de inicio del intervalo	Número que entra en el intervalo	Número de retirada durante el intervalo
		12	3	1
		18	2	1
		24	1	1
	PD	0	40	0
		6	6	0
		12	1	0

Tabla de mortalidad

Controles de primer orden	Hora de inicio del intervalo	Número expuesto a riesgo	Número de eventos terminales
	12	2,500	0
	18	1,500	0
	24	,500	0
PD	0	40,000	34
	6	6,000	5
	12	1,000	1

Tabla de mortalidad

Controles de primer orden	Hora de inicio del intervalo	Proporción que termina	Proporción que sobrevive
	12	,00	1,00
	18	,00	1,00
	24	,00	1,00
PD	0	,85	,15
	6	,83	,17
	12	1,00	,00

Tabla de mortalidad

Controles de primer orden	Hora de inicio del intervalo	Proporción acumulada que sobrevive al final del intervalo	Error estándar de la proporción acumulada que perdura al final del intervalo
	12	,43	,19
	18	,43	,19
	24	,43	,19
PD	0	,15	,06
	6	,02	,02
	12	,00	,00

Tabla de mortalidad

Controles de primer orden		Hora de inicio del intervalo	Densidad de probabilidad	Error estándar de la densidad de probabilidad
		12	,000	,000
		18	,000	,000
		24	,000	,000
	PD	0	,142	,009
		6	,021	,009
		12	,004	,004

Tabla de mortalidad

Controles de primer orden		Hora de inicio del intervalo	Índice de riesgo	Error estándar del índice de riesgo
		12	,00	,00
		18	,00	,00
		24	,00	,00
	PD	0	,25	,03
		6	,24	,07
		12	,33	,00

Kaplan-Meier OS AND CR-PR VS SD-PD

Resumen de procesamiento de casos

RC y RP vs SD/EP	N total	N de eventos	Censurado	
			N	Porcentaje
1,00	16	0	16	100,0%
2,00	50	45	5	10,0%
Global	66	45	21	31,8%

Comparaciones globales

	Chi-cuadrado	gl	Sig.
Log Rank (Mantel-Cox)	33,408	1	,000

Prueba de igualdad de distribuciones de supervivencia para los distintos niveles de RC y RP vs SD/EP.

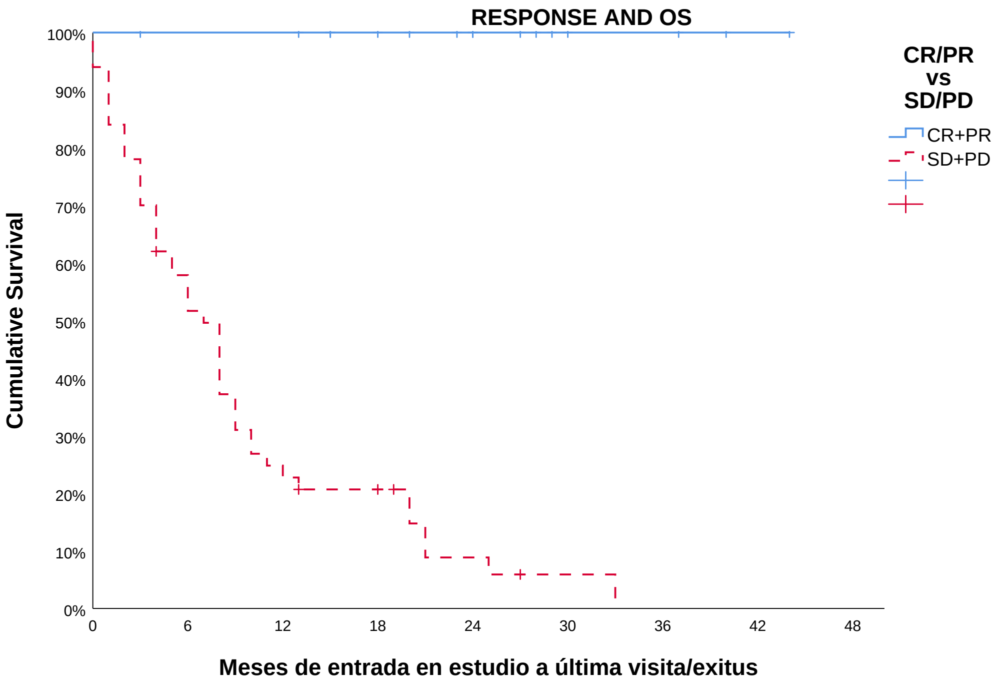

Análisis de supervivencia

Variable de supervivencia : Meses de entrada en estudio a última vis ita/exitus

Tabla de mortalidad

Controles de primer orden		Hora de inicio del intervalo	Número que entra en el intervalo	Número de retirada durante el intervalo
RC y RP vs SD/EP	1,00	0	16	1
		6	15	0
		12	15	3
		18	12	3
		24	9	5
		30	4	1
		36	3	2
		42	1	1
	2,00	0	50	1
		6	28	0
		12	12	1
		18	9	2
		24	3	1
		30	1	0

Tabla de mortalidad

Controles de primer orden		Hora de inicio del intervalo	Número expuesto a riesgo	Número de eventos terminales
RC y RP vs SD/EP	1,00	0	15,500	0
		6	15,000	0
		12	13,500	0
		18	10,500	0
		24	6,500	0
		30	3,500	0
		36	2,000	0
		42	,500	0
	2,00	0	49,500	21
		6	28,000	16
		12	11,500	2
		18	8,000	4
		24	2,500	1
		30	1,000	1

Tabla de mortalidad

Controles de primer orden		Hora de inicio del intervalo	Proporción que termina	Proporción que sobrevive
RC y RP vs SD/EP	1,00	0	,00	1,00
		6	,00	1,00
		12	,00	1,00
		18	,00	1,00
		24	,00	1,00
		30	,00	1,00
		36	,00	1,00
		42	,00	1,00
	2,00	0	,42	,58
		6	,57	,43
		12	,17	,83
		18	,50	,50
		24	,40	,60
		30	1,00	,00

Tabla de mortalidad

Controles de primer orden		Hora de inicio del intervalo	Proporción acumulada que sobrevive al final del intervalo	Error estándar de la proporción acumulada que perdura al final del intervalo
RC y RP vs SD/EP	1,00	0	1,00	,00
		6	1,00	,00
		12	1,00	,00
		18	1,00	,00
		24	1,00	,00
		30	1,00	,00
		36	1,00	,00
		42	1,00	,00
	2,00	0	,58	,07
		6	,25	,06
		12	,20	,06
		18	,10	,05
		24	,06	,04
		30	,00	,00

Tabla de mortalidad

Controles de primer orden		Hora de inicio del intervalo	Densidad de probabilidad	Error estándar de la densidad de probabilidad
RC y RP vs SD/EP	1,00	0	,000	,000
		6	,000	,000
		12	,000	,000
		18	,000	,000
		24	,000	,000
		30	,000	,000
		36	,000	,000
		42	,000	,000
	2,00	0	,071	,012
		6	,055	,011
		12	,007	,005
		18	,017	,008
		24	,007	,006
		30	,010	,007

Tabla de mortalidad

Controles de primer orden		Hora de inicio del intervalo	Índice de riesgo	Error estándar del índice de riesgo
RC y RP vs SD/EP	1,00	0	,00	,00
		6	,00	,00
		12	,00	,00
		18	,00	,00
		24	,00	,00
		30	,00	,00
		36	,00	,00
		42	,00	,00
	2,00	0	,09	,02
		6	,13	,03
		12	,03	,02
		18	,11	,05
		24	,08	,08
		30	,33	,00

Kaplan-Meier EFS CR-PR VS SD-PD

Resumen de procesamiento de casos

RC y RP vs SD/EP	N total	N de eventos	Censurado	
			N	Porcentaje
1,00	16	5	11	68,8%
2,00	50	47	3	6,0%
Global	66	52	14	21,2%

Medias y medianas para el tiempo de supervivencia

RC y RP vs SD/EP	Media[a]				Mediana
			Intervalo de confianza de 95 %		
	Estimación	Desv. Error	Límite inferior	Límite superior	Estimación
1,00	30,715	3,274	24,298	37,131	.
2,00	5,808	1,020	3,810	7,807	3,000
Global	11,954	1,778	8,469	15,438	4,000

Medias y medianas para el tiempo de supervivencia

RC y RP vs SD/EP	Mediana		
		Intervalo de confianza de 95 %	
	Desv. Error	Límite inferior	Límite superior
1,00	.	.	.
2,00	,436	2,145	3,855
Global	1,487	1,086	6,914

a. La estimación está limitada al tiempo de supervivencia más largo, si está censurado.

Comparaciones globales

	Chi-cuadrado	gl	Sig.
Log Rank (Mantel-Cox)	27,690	1	,000

Prueba de igualdad de distribuciones de supervivencia para los distintos niveles de RC y RP vs SD/EP.

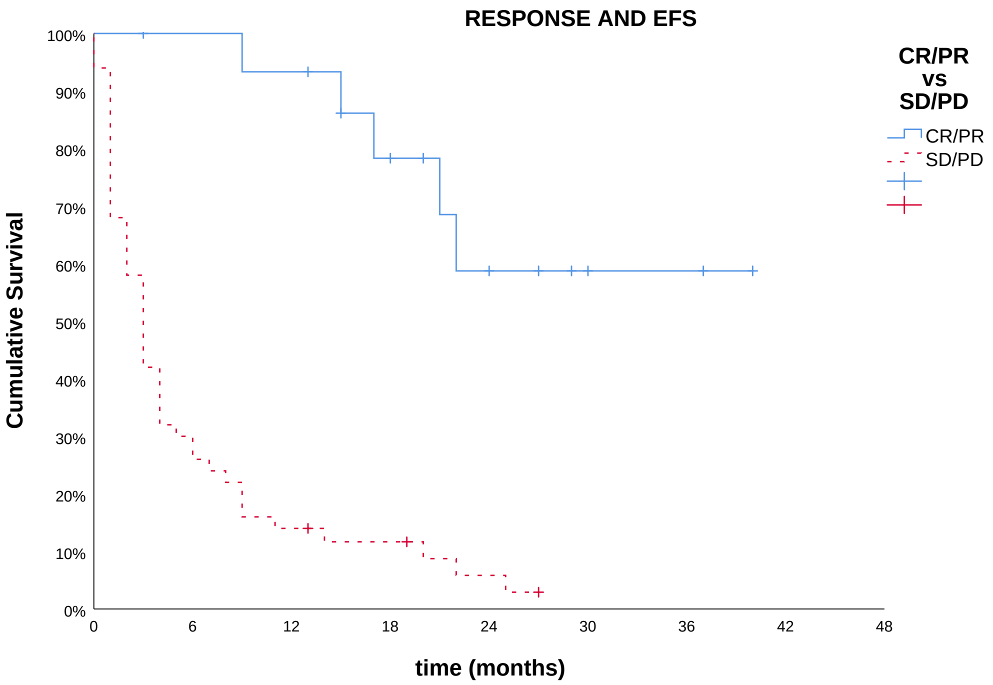

Análisis de supervivencia

Variable de supervivencia : SLE (meses, episodios)

Tabla de mortalidad

Controles de primer orden		Hora de inicio del intervalo	Número que entra en el intervalo	Número de retirada durante el intervalo
RC y RP vs SD/EP	1,00	0	16	1
		6	15	0
		12	14	2
		18	10	2
		24	6	3
		30	3	1
		36	2	2
	2,00	0	47	0
		6	12	0
		12	4	1
		18	2	1
		24	1	1

Tabla de mortalidad

Controles de primer orden		Hora de inicio del intervalo	Número expuesto a riesgo	Número de eventos terminales
RC y RP vs SD/EP	1,00	0	15,500	0
		6	15,000	1
		12	13,000	2
		18	9,000	2
		24	4,500	0
		30	2,500	0
		36	1,000	0
	2,00	0	47,000	35
		6	12,000	8
		12	3,500	1
		18	1,500	0
		24	,500	0

Tabla de mortalidad

Controles de primer orden		Hora de inicio del intervalo	Proporción que termina	Proporción que sobrevive
RC y RP vs SD/EP	1,00	0	,00	1,00
		6	,07	,93
		12	,15	,85
		18	,22	,78
		24	,00	1,00
		30	,00	1,00
		36	,00	1,00
	2,00	0	,74	,26
		6	,67	,33
		12	,29	,71
		18	,00	1,00
		24	,00	1,00

Tabla de mortalidad

Controles de primer orden		Hora de inicio del intervalo	Proporción acumulada que sobrevive al final del intervalo	Error estándar de la proporción acumulada que perdura al final del intervalo
RC y RP vs SD/EP	1,00	0	1,00	,00
		6	,93	,06
		12	,79	,11
		18	,61	,14
		24	,61	,14
		30	,61	,14
		36	,61	,14
	2,00	0	,26	,06
		6	,09	,04
		12	,06	,04
		18	,06	,04
		24	,06	,04

Tabla de mortalidad

Controles de primer orden		Hora de inicio del intervalo	Densidad de probabilidad	Error estándar de la densidad de probabilidad
RC y RP vs SD/EP	1,00	0	,000	,000
		6	,011	,011
		12	,024	,016
		18	,029	,019
		24	,000	,000
		30	,000	,000
		36	,000	,000
	2,00	0	,124	,011
		6	,028	,009
		12	,004	,004
		18	,000	,000
		24	,000	,000

Tabla de mortalidad

Controles de primer orden		Hora de inicio del intervalo	Índice de riesgo	Error estándar del índice de riesgo
RC y RP vs SD/EP	1,00	0	,00	,00
		6	,01	,01
		12	,03	,02
		18	,04	,03
		24	,00	,00
		30	,00	,00
		36	,00	,00
	2,00	0	,20	,03
		6	,17	,05
		12	,06	,05
		18	,00	,00
		24	,00	,00

Kaplan-Meier OS CR-PR-SD VS PD

Resumen de procesamiento de casos

RC,RP,SD VS EP	N total	N de eventos	Censurado	
			N	Porcentaje
CR+PR+SD	23	4	19	82,6%
PD	43	41	2	4,7%
Global	66	45	21	31,8%

Medias y medianas para el tiempo de supervivencia

RC,RP,SD VS EP	Media[a]				Mediana
			Intervalo de confianza de 95 %		
	Estimación	Desv. Error	Límite inferior	Límite superior	Estimación
CR+PR+SD	37,678	2,849	32,095	43,261	.
PD	7,750	1,209	5,381	10,119	6,000
Global	17,135	2,162	12,897	21,373	9,000

Medias y medianas para el tiempo de supervivencia

RC,RP,SD VS EP	Mediana		
		Intervalo de confianza de 95 %	
	Desv. Error	Límite inferior	Límite superior
CR+PR+SD	.	.	.
PD	1,257	3,536	8,464
Global	1,076	6,890	11,110

a. La estimación está limitada al tiempo de supervivencia más largo, si está censurado.

Comparaciones globales

	Chi-cuadrado	gl	Sig.
Log Rank (Mantel-Cox)	40,287	1	,000

Prueba de igualdad de distribuciones de supervivencia para los distintos niveles de RC,RP,SD VS EP.

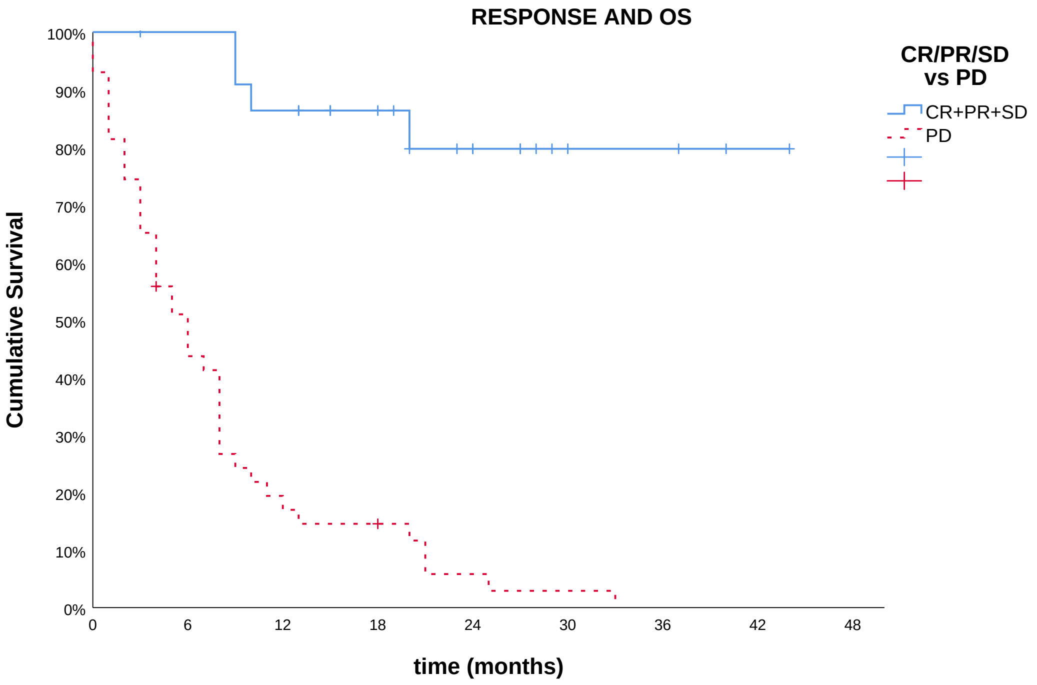

Análisis de supervivencia

Variable de supervivencia : Meses de entrada en estudio a última vis ita/exitus

Tabla de mortalidad

Controles de primer orden		Hora de inicio del intervalo	Número que entra en el intervalo	Número de retirada durante el intervalo
RC,RP,SD VS EP	CR+PR+SD	0	23	1
		6	22	0
		12	19	4
		18	15	4
		24	10	6
		30	4	1
		36	3	2
		42	1	1
	PD	0	43	1
		6	21	0
		12	8	0
		18	6	1
		24	2	0
		30	1	0

Tabla de mortalidad

Controles de primer orden		Hora de inicio del intervalo	Número expuesto a riesgo	Número de eventos terminales
RC,RP,SD VS EP	CR+PR+SD	0	22,500	0
		6	22,000	3
		12	17,000	0
		18	13,000	1
		24	7,000	0
		30	3,500	0
		36	2,000	0
		42	,500	0
	PD	0	42,500	21
		6	21,000	13
		12	8,000	2
		18	5,500	3
		24	2,000	1
		30	1,000	1

Tabla de mortalidad

Controles de primer orden		Hora de inicio del intervalo	Proporción que termina	Proporción que sobrevive
RC,RP,SD VS EP	CR+PR+SD	0	,00	1,00
		6	,14	,86
		12	,00	1,00
		18	,08	,92
		24	,00	1,00
		30	,00	1,00
		36	,00	1,00
		42	,00	1,00
	PD	0	,49	,51
		6	,62	,38
		12	,25	,75
		18	,55	,45
		24	,50	,50
		30	1,00	,00

Tabla de mortalidad

Controles de primer orden		Hora de inicio del intervalo	Proporción acumulada que sobrevive al final del intervalo	Error estándar de la proporción acumulada que perdura al final del intervalo
RC,RP,SD VS EP	CR+PR+SD	0	1,00	,00
		6	,86	,07
		12	,86	,07
		18	,80	,09
		24	,80	,09
		30	,80	,09
		36	,80	,09
		42	,80	,09
	PD	0	,51	,08
		6	,19	,06
		12	,14	,05
		18	,07	,04
		24	,03	,03
		30	,00	,00

Tabla de mortalidad

Controles de primer orden		Hora de inicio del intervalo	Densidad de probabilidad	Error estándar de la densidad de probabilidad
RC,RP,SD VS EP	CR+PR+SD	0	,000	,000
		6	,023	,012
		12	,000	,000
		18	,011	,011
		24	,000	,000
		30	,000	,000
		36	,000	,000
		42	,000	,000
	PD	0	,082	,013
		6	,052	,012
		12	,008	,006
		18	,013	,007
		24	,005	,005
		30	,005	,005

Tabla de mortalidad

Controles de primer orden		Hora de inicio del intervalo	Índice de riesgo	Error estándar del índice de riesgo
RC,RP,SD VS EP	CR+PR+SD	0	,00	,00
		6	,02	,01
		12	,00	,00
		18	,01	,01
		24	,00	,00
		30	,00	,00
		36	,00	,00
		42	,00	,00
	PD	0	,11	,02
		6	,15	,04
		12	,05	,03
		18	,13	,07
		24	,11	,10
		30	,33	,00

Kaplan-Meier EFS AND CR-PR-SD VS PD

Resumen de procesamiento de casos

RC,RP,SD VS EP	N total	N de eventos	Censurado	
			N	Porcentaje
CR+PR+SD	23	9	14	60,9%
PD	43	43	0	0,0%
Global	66	52	14	21,2%

Medias y medianas para el tiempo de supervivencia

RC,RP,SD VS EP	Media[a]				Mediana
	Estimación	Desv. Error	Intervalo de confianza de 95 %		Estimación
			Límite inferior	Límite superior	
CR+PR+SD	27,290	3,192	21,034	33,547	.
PD	4,419	,885	2,684	6,153	3,000
Global	11,954	1,778	8,469	15,438	4,000

Medias y medianas para el tiempo de supervivencia

RC,RP,SD VS EP	Mediana		
	Desv. Error	Intervalo de confianza de 95 %	
		Límite inferior	Límite superior
CR+PR+SD	.	.	.
PD	,473	2,073	3,927
Global	1,487	1,086	6,914

a. La estimación está limitada al tiempo de supervivencia más largo, si está censurado.

Comparaciones globales

	Chi-cuadrado	gl	Sig.
Log Rank (Mantel-Cox)	39,813	1	,000

Prueba de igualdad de distribuciones de supervivencia para los distintos niveles de RC,RP,SD VS EP.

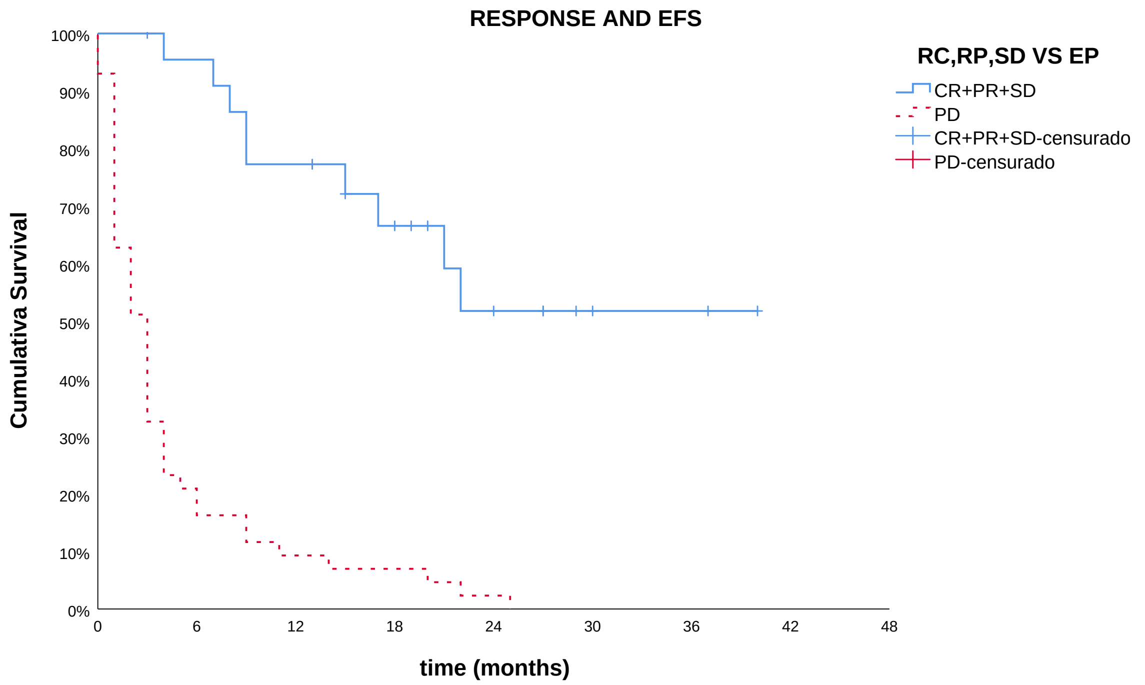

Análisis de supervivencia

Variable de supervivencia : SLE (meses, episodios)

Tabla de mortalidad

Controles de primer orden		Hora de inicio del intervalo	Número que entra en el intervalo	Número de retirada durante el intervalo
RC,RP,SD VS EP	CR+PR+SD	0	23	1
		6	21	0
		12	17	3
		18	12	3
		24	7	4
		30	3	1
		36	2	2
	PD	0	40	0
		6	6	0
		12	1	0

Tabla de mortalidad

Controles de primer orden		Hora de inicio del intervalo	Número expuesto a riesgo	Número de eventos terminales
RC,RP,SD VS EP	CR+PR+SD	0	22,500	1
		6	21,000	4
		12	15,500	2
		18	10,500	2
		24	5,000	0
		30	2,500	0
		36	1,000	0
	PD	0	40,000	34
		6	6,000	5
		12	1,000	1

Tabla de mortalidad

Controles de primer orden		Hora de inicio del intervalo	Proporción que termina	Proporción que sobrevive
RC,RP,SD VS EP	CR+PR+SD	0	,04	,96
		6	,19	,81
		12	,13	,87
		18	,19	,81
		24	,00	1,00
		30	,00	1,00
		36	,00	1,00
	PD	0	,85	,15
		6	,83	,17
		12	1,00	,00

Tabla de mortalidad

Controles de primer orden		Hora de inicio del intervalo	Proporción acumulada que sobrevive al final del intervalo	Error estándar de la proporción acumulada que perdura al final del intervalo
RC,RP,SD VS EP	CR+PR+SD	0	,96	,04
		6	,77	,09
		12	,67	,10
		18	,55	,12
		24	,55	,12
		30	,55	,12
		36	,55	,12
	PD	0	,15	,06
		6	,02	,02
		12	,00	,00

Tabla de mortalidad

Controles de primer orden		Hora de inicio del intervalo	Densidad de probabilidad	Error estándar de la densidad de probabilidad
RC,RP,SD VS EP	CR+PR+SD	0	,007	,007
		6	,030	,014
		12	,017	,011
		18	,021	,014
		24	,000	,000
		30	,000	,000
		36	,000	,000
	PD	0	,142	,009
		6	,021	,009
		12	,004	,004

Tabla de mortalidad

Controles de primer orden		Hora de inicio del intervalo	Índice de riesgo	Error estándar del índice de riesgo
RC,RP,SD VS EP	CR+PR+SD	0	,01	,01
		6	,04	,02
		12	,02	,02
		18	,04	,02
		24	,00	,00
		30	,00	,00
		36	,00	,00
	PD	0	,25	,03
		6	,24	,07
		12	,33	,00

Kaplan-Meier OS AND IMMUNOTHERAPY

Resumen de procesamiento de casos

INMUNO	N total	N de eventos	Censurado	
			N	Porcentaje
YES	31	16	15	48,4%
NO	36	29	7	19,4%
Global	67	45	22	32,8%

Medias y medianas para el tiempo de supervivencia

INMUNO	Media[a]				Mediana	
			Intervalo de confianza de 95 %			
	Estimación	Desv. Error	Límite inferior	Límite superior	Estimación	Desv. Error
YES	22,600	2,515	17,671	27,529	25,000	4,402
NO	11,081	2,470	6,240	15,922	6,000	1,842
Global	17,715	2,170	13,461	21,969	9,000	1,327

Medias y medianas para el tiempo de supervivencia

	Mediana	
	Intervalo de confianza de 95 %	
INMUNO	Límite inferior	Límite superior
YES	16,372	33,628
NO	2,390	9,610
Global	6,399	11,601

a. La estimación está limitada al tiempo de supervivencia más largo, si está censurado.

Comparaciones globales

	Chi-cuadrado	gl	Sig.
Log Rank (Mantel-Cox)	13,052	1	,000

Prueba de igualdad de distribuciones de supervivencia para los distintos niveles de INMUNO.

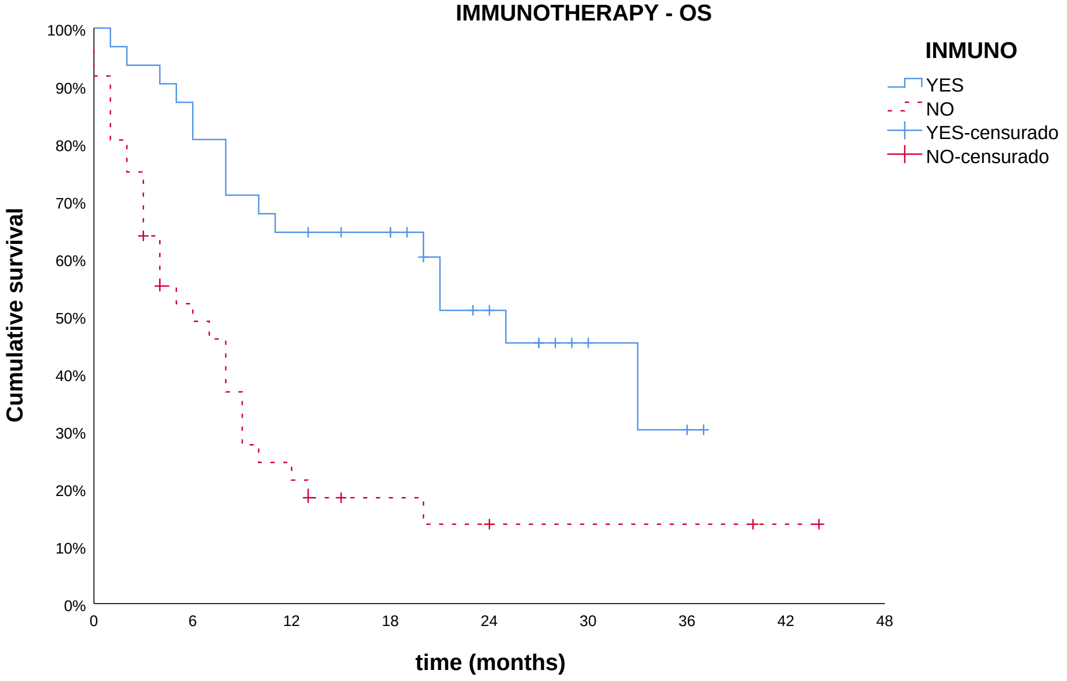

Análisis de supervivencia

Variable de supervivencia : Meses de entrada en estudio a última vis ita/exitus

Tabla de mortalidad

Controles de primer orden		Hora de inicio del intervalo	Número que entra en el intervalo	Número de retirada durante el intervalo
INMUNO	YES	0	31	0
		6	27	0
		12	20	2
		18	18	5
		24	10	5
		30	4	1
		36	2	2
	NO	0	36	2
		6	17	0
		12	8	2
		18	4	0
		24	3	1
		30	2	0
		36	2	1
		42	1	1

Tabla de mortalidad

Controles de primer orden		Hora de inicio del intervalo	Número expuesto a riesgo	Número de eventos terminales
INMUNO	YES	0	31,000	4
		6	27,000	7
		12	19,000	0
		18	15,500	3
		24	7,500	1
		30	3,500	1
		36	1,000	0
	NO	0	35,000	17
		6	17,000	9
		12	7,000	2
		18	4,000	1
		24	2,500	0
		30	2,000	0
		36	1,500	0
		42	,500	0

Tabla de mortalidad

Controles de primer orden		Hora de inicio del intervalo	Proporción que termina	Proporción que sobrevive
INMUNO	YES	0	,13	,87
		6	,26	,74
		12	,00	1,00
		18	,19	,81
		24	,13	,87
		30	,29	,71
		36	,00	1,00
	NO	0	,49	,51
		6	,53	,47
		12	,29	,71
		18	,25	,75
		24	,00	1,00
		30	,00	1,00
		36	,00	1,00
		42	,00	1,00

Tabla de mortalidad

Controles de primer orden		Hora de inicio del intervalo	Proporción acumulada que sobrevive al final del intervalo	Error estándar de la proporción acumulada que perdura al final del intervalo
INMUNO	YES	0	,87	,06
		6	,65	,09
		12	,65	,09
		18	,52	,09
		24	,45	,10
		30	,32	,13
		36	,32	,13
	NO	0	,51	,08
		6	,24	,07
		12	,17	,07
		18	,13	,06
		24	,13	,06
		30	,13	,06
		36	,13	,06
		42	,13	,06

Tabla de mortalidad

Controles de primer orden		Hora de inicio del intervalo	Densidad de probabilidad	Error estándar de la densidad de probabilidad
INMUNO	YES	0	,022	,010
		6	,038	,013
		12	,000	,000
		18	,021	,011
		24	,012	,011
		30	,021	,019
		36	,000	,000
	NO	0	,081	,014
		6	,045	,013
		12	,012	,008
		18	,007	,007
		24	,000	,000
		30	,000	,000
		36	,000	,000
		42	,000	,000

Tabla de mortalidad

Controles de primer orden		Hora de inicio del intervalo	Índice de riesgo	Error estándar del índice de riesgo
INMUNO	YES	0	,02	,01
		6	,05	,02
		12	,00	,00
		18	,04	,02
		24	,02	,02
		30	,06	,05
		36	,00	,00
	NO	0	,11	,02
		6	,12	,04
		12	,06	,04
		18	,05	,05
		24	,00	,00
		30	,00	,00
		36	,00	,00
		42	,00	,00

Kaplan-Meier EFS AND IMMUNOTHERAPY

Resumen de procesamiento de casos

INMUNO	N total	N de eventos	Censurado	
			N	Porcentaje
YES	31	20	11	35,5%
NO	36	33	3	8,3%
Global	67	53	14	20,9%

Medias y medianas para el tiempo de supervivencia

INMUNO	Media[a]				Mediana	
			Intervalo de confianza de 95 %			
	Estimación	Desv. Error	Límite inferior	Límite superior	Estimación	Desv. Error
YES	18,096	2,554	13,091	23,101	20,000	6,959
NO	5,881	1,506	2,929	8,833	3,000	,592
Global	12,035	1,734	8,636	15,434	4,000	1,499

Medias y medianas para el tiempo de supervivencia

INMUNO	Mediana	
	Intervalo de confianza de 95 %	
	Límite inferior	Límite superior
YES	6,360	33,640
NO	1,840	4,160
Global	1,061	6,939

a. La estimación está limitada al tiempo de supervivencia más largo, si está censurado.

Comparaciones globales

	Chi-cuadrado	gl	Sig.
Log Rank (Mantel-Cox)	15,607	1	,000

Prueba de igualdad de distribuciones de supervivencia para los distintos niveles de INMUNO.

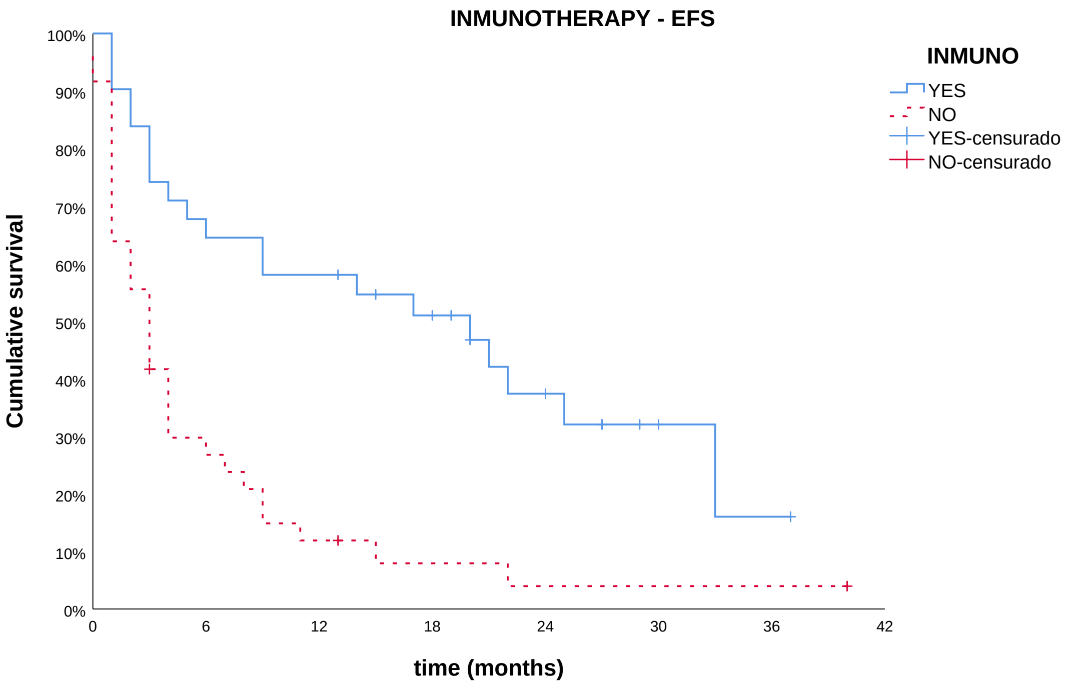

Análisis de supervivencia

Variable de supervivencia : SLE (meses, episodios)

Tabla de mortalidad

Controles de primer orden		Hora de inicio del intervalo	Número que entra en el intervalo	Número de retirada durante el intervalo
INMUNO	YES	0	28	0
		6	18	0
		12	15	2
		18	11	3
		24	7	4
		30	3	1
		36	1	1
	NO	0	36	1
		6	10	0
		12	4	1
		18	2	0
		24	1	0
		30	1	0
		36	1	1

Tabla de mortalidad

Controles de primer orden		Hora de inicio del intervalo	Número expuesto a riesgo	Número de eventos terminales
INMUNO	YES	0	28,000	10
		6	18,000	3
		12	14,000	2
		18	9,500	1
		24	5,000	0
		30	2,500	1
		36	,500	0
	NO	0	35,500	25
		6	10,000	6
		12	3,500	1
		18	2,000	1
		24	1,000	0
		30	1,000	0
		36	,500	0

Tabla de mortalidad

Controles de primer orden		Hora de inicio del intervalo	Proporción que termina	Proporción que sobrevive
INMUNO	YES	0	,36	,64
		6	,17	,83
		12	,14	,86
		18	,11	,89
		24	,00	1,00
		30	,40	,60
		36	,00	1,00
	NO	0	,70	,30
		6	,60	,40
		12	,29	,71
		18	,50	,50
		24	,00	1,00
		30	,00	1,00
		36	,00	1,00

Tabla de mortalidad

Controles de primer orden		Hora de inicio del intervalo	Proporción acumulada que sobrevive al final del intervalo	Error estándar de la proporción acumulada que perdura al final del intervalo
INMUNO	YES	0	,64	,09
		6	,54	,09
		12	,46	,10
		18	,41	,10
		24	,41	,10
		30	,25	,14
		36	,25	,14
	NO	0	,30	,08
		6	,12	,06
		12	,08	,05
		18	,04	,04
		24	,04	,04
		30	,04	,04
		36	,04	,04

Tabla de mortalidad

Controles de primer orden		Hora de inicio del intervalo	Densidad de probabilidad	Error estándar de la densidad de probabilidad
INMUNO	YES	0	,060	,015
		6	,018	,010
		12	,013	,009
		18	,008	,008
		24	,000	,000
		30	,027	,022
		36	,000	,000
	NO	0	,117	,013
		6	,030	,011
		12	,006	,005
		18	,007	,006
		24	,000	,000
		30	,000	,000
		36	,000	,000

Tabla de mortalidad

Controles de primer orden		Hora de inicio del intervalo	Índice de riesgo	Error estándar del índice de riesgo
INMUNO	YES	0	,07	,02
		6	,03	,02
		12	,03	,02
		18	,02	,02
		24	,00	,00
		30	,08	,08
		36	,00	,00
	NO	0	,18	,03
		6	,14	,05
		12	,06	,05
		18	,11	,10
		24	,00	,00
		30	,00	,00
		36	,00	,00

Kaplan-Meier OS AND HDC

Resumen de procesamiento de casos

TPH	N total	N de eventos	Censurado	
			N	Porcentaje
YES	14	3	11	78,6%
NO	52	41	11	21,2%
Global	66	44	22	33,3%

Medias y medianas para el tiempo de supervivencia

TPH	Media[a]				Mediana	
	Estimación	Desv. Error	Intervalo de confianza de 95 %		Estimación	Desv. Error
			Límite inferior	Límite superior		
YES	30,385	3,361	23,797	36,972	.	.
NO	13,945	2,148	9,735	18,154	8,000	1,170
Global	17,809	2,203	13,491	22,126	9,000	1,076

Medias y medianas para el tiempo de supervivencia

	Mediana	
	Intervalo de confianza de 95 %	
TPH	Límite inferior	Límite superior
YES	.	.
NO	5,706	10,294
Global	6,890	11,110

a. La estimación está limitada al tiempo de supervivencia más largo, si está censurado.

Comparaciones globales

	Chi-cuadrado	gl	Sig.
Log Rank (Mantel-Cox)	10,567	1	,001

Prueba de igualdad de distribuciones de supervivencia para los distintos niveles de TPH .

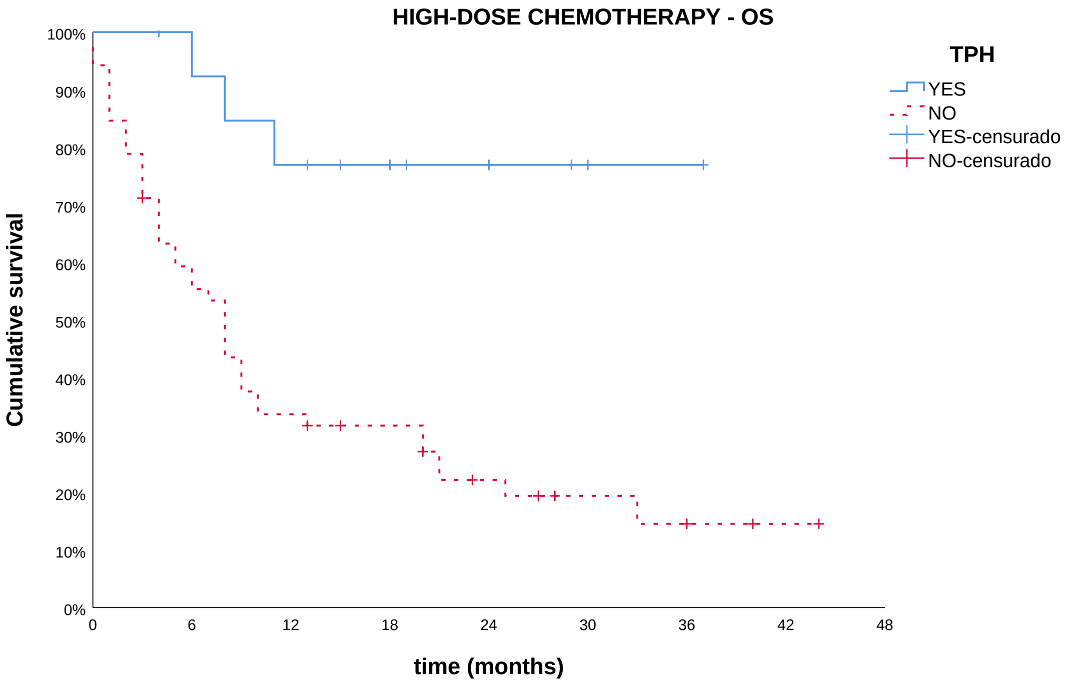

Análisis de supervivencia

Variable de supervivencia : Meses de entrada en estudio a última vis ita/exitus

Tabla de mortalidad

Controles de primer orden		Hora de inicio del intervalo	Número que entra en el intervalo	Número de retirada durante el intervalo
TPH	YES	0	14	1
		6	13	0
		12	10	2
		18	8	3
		24	5	3
		30	2	1
		36	1	1
	NO	0	52	1
		6	30	0
		12	17	2
		18	14	2
		24	8	3
		30	4	0
		36	3	2
		42	1	1

Tabla de mortalidad

Controles de primer orden		Hora de inicio del intervalo	Número expuesto a riesgo	Número de eventos terminales
TPH	YES	0	13,500	0
		6	13,000	3
		12	9,000	0
		18	6,500	0
		24	3,500	0
		30	1,500	0
		36	,500	0
	NO	0	51,500	21
		6	30,000	13
		12	16,000	1
		18	13,000	4
		24	6,500	1
		30	4,000	1
		36	2,000	0
		42	,500	0

Tabla de mortalidad

Controles de primer orden		Hora de inicio del intervalo	Proporción que termina	Proporción que sobrevive
TPH	YES	0	,00	1,00
		6	,23	,77
		12	,00	1,00
		18	,00	1,00
		24	,00	1,00
		30	,00	1,00
		36	,00	1,00
	NO	0	,41	,59
		6	,43	,57
		12	,06	,94
		18	,31	,69
		24	,15	,85
		30	,25	,75
		36	,00	1,00
		42	,00	1,00

Tabla de mortalidad

Controles de primer orden		Hora de inicio del intervalo	Proporción acumulada que sobrevive al final del intervalo	Error estándar de la proporción acumulada que perdura al final del intervalo
TPH	YES	0	1,00	,00
		6	,77	,12
		12	,77	,12
		18	,77	,12
		24	,77	,12
		30	,77	,12
		36	,77	,12
	NO	0	,59	,07
		6	,34	,07
		12	,31	,07
		18	,22	,06
		24	,18	,06
		30	,14	,06
		36	,14	,06
		42	,14	,06

Tabla de mortalidad

Controles de primer orden		Hora de inicio del intervalo	Densidad de probabilidad	Error estándar de la densidad de probabilidad
TPH	YES	0	,000	,000
		6	,038	,019
		12	,000	,000
		18	,000	,000
		24	,000	,000
		30	,000	,000
		36	,000	,000
	NO	0	,068	,011
		6	,043	,010
		12	,003	,003
		18	,016	,008
		24	,006	,005
		30	,008	,007
		36	,000	,000
		42	,000	,000

Tabla de mortalidad

Controles de primer orden		Hora de inicio del intervalo	Índice de riesgo	Error estándar del índice de riesgo
TPH	YES	0	,00	,00
		6	,04	,02
		12	,00	,00
		18	,00	,00
		24	,00	,00
		30	,00	,00
		36	,00	,00
	NO	0	,09	,02
		6	,09	,02
		12	,01	,01
		18	,06	,03
		24	,03	,03
		30	,05	,05
		36	,00	,00
		42	,00	,00

Kaplan-Meier EFS AND HDC

Medias y medianas para el tiempo de supervivencia

	Media[a]				Mediana	
			Intervalo de confianza de 95 %			
TPH	Estimación	Desv. Error	Límite inferior	Límite superior	Estimación	Desv. Error
YES	23,786	4,062	15,825	31,747	.	.
NO	9,050	1,612	5,891	12,209	3,000	,645
Global	12,187	1,754	8,750	15,624	4,000	1,764

Medias y medianas para el tiempo de supervivencia

	Mediana	
	Intervalo de confianza de 95 %	
TPH	Límite inferior	Límite superior
YES	.	.
NO	1,735	4,265
Global	,542	7,458

a. La estimación está limitada al tiempo de supervivencia más largo, si está censurado.

Comparaciones globales

	Chi-cuadrado	gl	Sig.
Log Rank (Mantel-Cox)	10,456	1	,001

Prueba de igualdad de distribuciones de supervivencia para los distintos niveles de TPH .

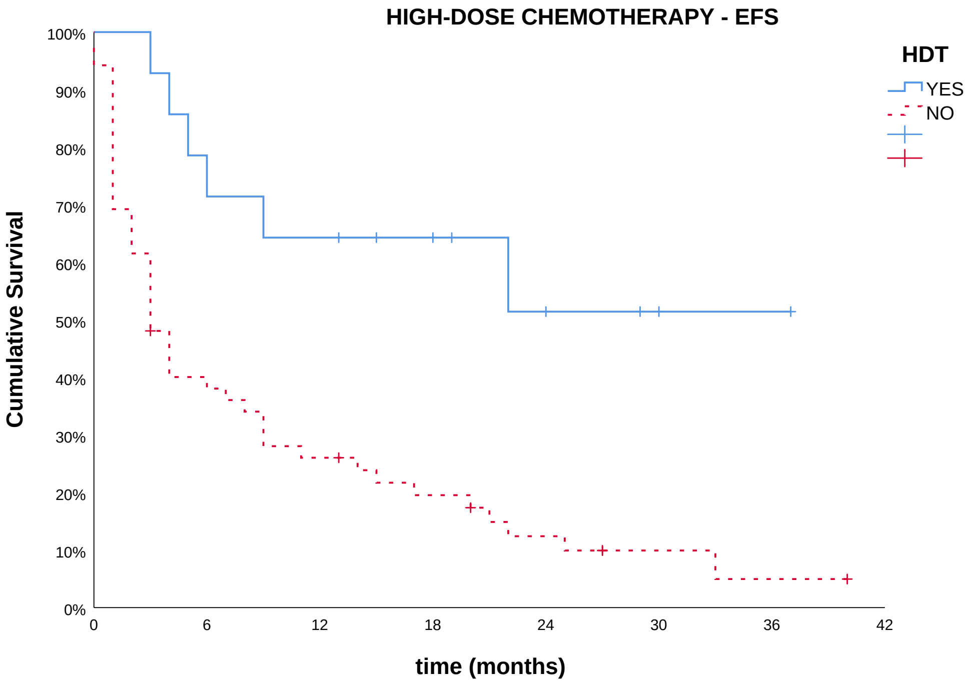

Análisis de supervivencia

Variable de supervivencia : SLE (meses, episodios)

Tabla de mortalidad

Controles de primer orden		Hora de inicio del intervalo	Número que entra en el intervalo	Número de retirada durante el intervalo
TPH	YES	0	14	0
		6	11	0
		12	9	2
		18	7	2
		24	4	2
		30	2	1
		36	1	1
	NO	0	49	1
		6	17	0
		12	10	1
		18	6	1
		24	4	2
		30	2	0
		36	1	1

Tabla de mortalidad

Controles de primer orden		Hora de inicio del intervalo	Número expuesto a riesgo	Número de eventos terminales
TPH	YES	0	14,000	3
		6	11,000	2
		12	8,000	0
		18	6,000	1
		24	3,000	0
		30	1,500	0
		36	,500	0
	NO	0	48,500	31
		6	17,000	7
		12	9,500	3
		18	5,500	1
		24	3,000	0
		30	2,000	1
		36	,500	0

Tabla de mortalidad

Controles de primer orden		Hora de inicio del intervalo	Proporción que termina	Proporción que sobrevive
TPH	YES	0	,21	,79
		6	,18	,82
		12	,00	1,00
		18	,17	,83
		24	,00	1,00
		30	,00	1,00
		36	,00	1,00
	NO	0	,64	,36
		6	,41	,59
		12	,32	,68
		18	,18	,82
		24	,00	1,00
		30	,50	,50
		36	,00	1,00

Tabla de mortalidad

Controles de primer orden		Hora de inicio del intervalo	Proporción acumulada que sobrevive al final del intervalo	Error estándar de la proporción acumulada que perdura al final del intervalo
TPH	YES	0	,79	,11
		6	,64	,13
		12	,64	,13
		18	,54	,14
		24	,54	,14
		30	,54	,14
		36	,54	,14
	NO	0	,36	,07
		6	,21	,06
		12	,15	,05
		18	,12	,05
		24	,12	,05
		30	,06	,05
		36	,06	,05

Tabla de mortalidad

Controles de primer orden		Hora de inicio del intervalo	Densidad de probabilidad	Error estándar de la densidad de probabilidad
TPH	YES	0	,036	,018
		6	,024	,016
		12	,000	,000
		18	,018	,017
		24	,000	,000
		30	,000	,000
		36	,000	,000
	NO	0	,107	,011
		6	,025	,009
		12	,011	,006
		18	,004	,004
		24	,000	,000
		30	,010	,008
		36	,000	,000

Tabla de mortalidad

Controles de primer orden		Hora de inicio del intervalo	Índice de riesgo	Error estándar del índice de riesgo
TPH	YES	0	,04	,02
		6	,03	,02
		12	,00	,00
		18	,03	,03
		24	,00	,00
		30	,00	,00
		36	,00	,00
	NO	0	,16	,02
		6	,09	,03
		12	,06	,04
		18	,03	,03
		24	,00	,00
		30	,11	,10
		36	,00	,00

Kaplan-Meier OS ALL CASES: SURGERY AND RADIOTHERAPY VS REST

Resumen de procesamiento de casos

LOC_COMB_VS	N total	N de eventos	Censurado	
			N	Porcentaje
CIRUGÍA + RADIOTERAPIA	10	3	7	70,0%
RESTO	54	40	14	25,9%
Global	64	43	21	32,8%

Medias y medianas para el tiempo de supervivencia

LOC_COMB_VS	Media[a]				Mediana
			Intervalo de confianza de 95 %		
	Estimación	Desv. Error	Límite inferior	Límite superior	Estimación
CIRUGÍA + RADIOTERAPIA	30,000	4,565	21,052	38,948	33,000
RESTO	14,737	2,250	10,327	19,148	8,000
Global	17,356	2,245	12,956	21,756	9,000

Medias y medianas para el tiempo de supervivencia

LOC_COMB_VS	Mediana		
		Intervalo de confianza de 95 %	
	Desv. Error	Límite inferior	Límite superior
CIRUGÍA + RADIOTERAPIA	20,288	,000	72,764
RESTO	,587	6,849	9,151
Global	1,164	6,718	11,282

a. La estimación está limitada al tiempo de supervivencia más largo, si está censurado.

Comparaciones globales

	Chi-cuadrado	gl	Sig.
Log Rank (Mantel-Cox)	6,605	1	,010

Prueba de igualdad de distribuciones de supervivencia para los distintos niveles de LOC_COMB_VS.

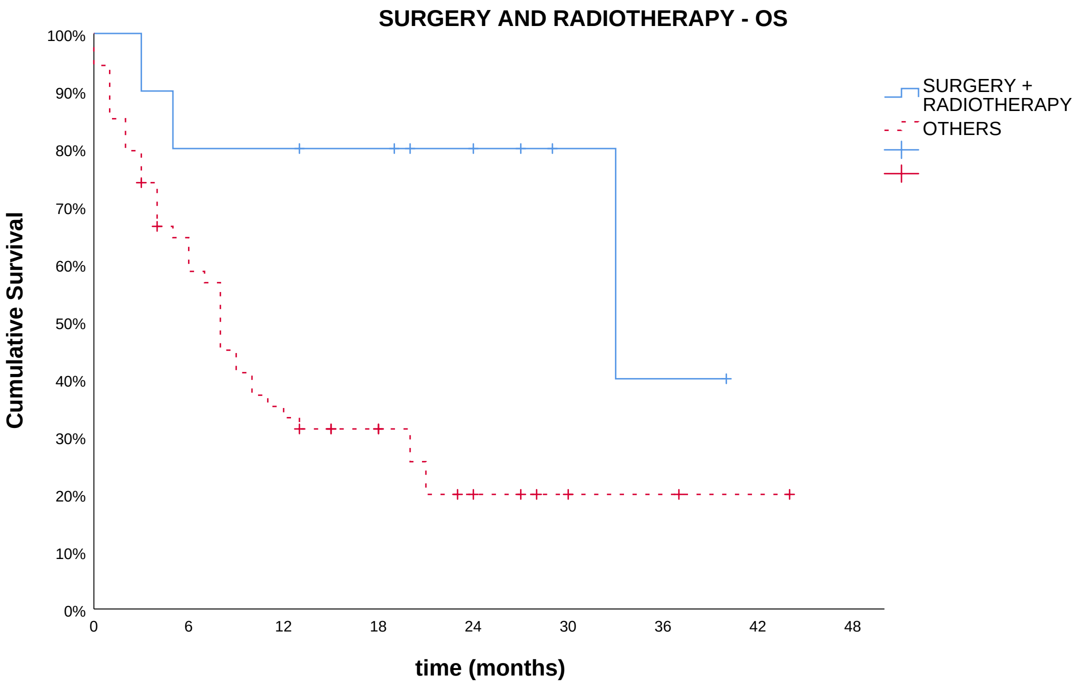

Análisis de supervivencia

Variable de supervivencia : Meses de entrada en estudio a última vis ita/exitus

Tabla de mortalidad

Controles de primer orden		Hora de inicio del intervalo	Número que entra en el intervalo
LOC_COMB_VS	CIRUGÍA + RADIOTERAPIA	0	10
		6	8
		12	8
		18	7
		24	5
		30	2
		36	1
	RESTO	0	54
		6	33
		12	18
		18	13
		24	6
		30	3
		36	2
		42	1

Tabla de mortalidad

Controles de primer orden		Hora de inicio del intervalo	Número de retirada durante el intervalo
LOC_COMB_VS	CIRUGÍA + RADIOTERAPIA	0	0
		6	0
		12	1
		18	2
		24	3
		30	0
		36	1
	RESTO	0	2
		6	0
		12	3
		18	3
		24	3
		30	1
		36	1
		42	1

Tabla de mortalidad

Controles de primer orden		Hora de inicio del intervalo	Número expuesto a riesgo
LOC_COMB_VS	CIRUGÍA + RADIOTERAPIA	0	10,000
		6	8,000
		12	7,500
		18	6,000
		24	3,500
		30	2,000
		36	,500
	RESTO	0	53,000
		6	33,000
		12	16,500
		18	11,500
		24	4,500
		30	2,500
		36	1,500
		42	,500

Tabla de mortalidad

Controles de primer orden		Hora de inicio del intervalo	Número de eventos terminales
LOC_COMB_VS	CIRUGÍA + RADIOTERAPIA	0	2
		6	0
		12	0
		18	0
		24	0
		30	1
		36	0
	RESTO	0	19
		6	15
		12	2
		18	4
		24	0
		30	0
		36	0
		42	0

Tabla de mortalidad

Controles de primer orden		Hora de inicio del intervalo	Proporción que termina
LOC_COMB_VS	CIRUGÍA + RADIOTERAPIA	0	,20
		6	,00
		12	,00
		18	,00
		24	,00
		30	,50
		36	,00
	RESTO	0	,36
		6	,45
		12	,12
		18	,35
		24	,00
		30	,00
		36	,00
		42	,00

Tabla de mortalidad

Controles de primer orden		Hora de inicio del intervalo	Proporción que sobrevive
LOC_COMB_VS	CIRUGÍA + RADIOTERAPIA	0	,80
		6	1,00
		12	1,00
		18	1,00
		24	1,00
		30	,50
		36	1,00
	RESTO	0	,64
		6	,55
		12	,88
		18	,65
		24	1,00
		30	1,00
		36	1,00
		42	1,00

Tabla de mortalidad

Controles de primer orden		Hora de inicio del intervalo	Proporción acumulada que sobrevive al final del intervalo
LOC_COMB_VS	CIRUGÍA + RADIOTERAPIA	0	,80
		6	,80
		12	,80
		18	,80
		24	,80
		30	,40
		36	,40
	RESTO	0	,64
		6	,35
		12	,31
		18	,20
		24	,20
		30	,20
		36	,20
		42	,20

Tabla de mortalidad

Controles de primer orden		Hora de inicio del intervalo	Error estándar de la proporción acumulada que perdura al final del intervalo
LOC_COMB_VS	CIRUGÍA + RADIOTERAPIA	0	,13
		6	,13
		12	,13
		18	,13
		24	,13
		30	,29
		36	,29
	RESTO	0	,07
		6	,07
		12	,06
		18	,06
		24	,06
		30	,06
		36	,06
		42	,06

Tabla de mortalidad

Controles de primer orden		Hora de inicio del intervalo	Densidad de probabilidad
LOC_COMB_VS	CIRUGÍA + RADIOTERAPIA	0	,033
		6	,000
		12	,000
		18	,000
		24	,000
		30	,067
		36	,000
	RESTO	0	,060
		6	,049
		12	,007
		18	,018
		24	,000
		30	,000
		36	,000
		42	,000

Tabla de mortalidad

Controles de primer orden		Hora de inicio del intervalo	Error estándar de la densidad de probabilidad
LOC_COMB_VS	CIRUGÍA + RADIOTERAPIA	0	,021
		6	,000
		12	,000
		18	,000
		24	,000
		30	,048
		36	,000
	RESTO	0	,011
		6	,011
		12	,005
		18	,008
		24	,000
		30	,000
		36	,000
		42	,000

Tabla de mortalidad

Controles de primer orden		Hora de inicio del intervalo	Índice de riesgo
LOC_COMB_VS	CIRUGÍA + RADIOTERAPIA	0	,04
		6	,00
		12	,00
		18	,00
		24	,00
		30	,11
		36	,00
	RESTO	0	,07
		6	,10
		12	,02
		18	,07
		24	,00
		30	,00
		36	,00
		42	,00

Tabla de mortalidad

Controles de primer orden		Hora de inicio del intervalo	Error estándar del índice de riesgo
LOC_COMB_VS	CIRUGÍA + RADIOTERAPIA	0	,03
		6	,00
		12	,00
		18	,00
		24	,00
		30	,10
		36	,00
	RESTO	0	,02
		6	,02
		12	,02
		18	,03
		24	,00
		30	,00
		36	,00
		42	,00

Kaplan-Meier EFS ALL CASES: SURGERY AND RADIOTHERAPY VS REST

Resumen de procesamiento de casos

LOC_COMB_VS	N total	N de eventos	Censurado N	Censurado Porcentaje
CIRUGÍA + RADIOTERAPIA	10	3	7	70,0%
RESTO	54	47	7	13,0%
Global	64	50	14	21,9%

Medias y medianas para el tiempo de supervivencia

	Media[a]				Mediana
			Intervalo de confianza de 95 %		
LOC_COMB_VS	Estimación	Desv. Error	Límite inferior	Límite superior	Estimación
CIRUGÍA + RADIOTERAPIA	29,620	5,003	19,815	39,425	.
RESTO	8,405	1,482	5,502	11,309	3,000
Global	11,990	1,838	8,388	15,591	4,000

Medias y medianas para el tiempo de supervivencia

	Mediana		
		Intervalo de confianza de 95 %	
LOC_COMB_VS	Desv. Error	Límite inferior	Límite superior
CIRUGÍA + RADIOTERAPIA	.	.	.
RESTO	,659	1,709	4,291
Global	1,055	1,932	6,068

a. La estimación está limitada al tiempo de supervivencia más largo, si está censurado.

Comparaciones globales

	Chi-cuadrado	gl	Sig.
Log Rank (Mantel-Cox)	11,835	1	,001

Prueba de igualdad de distribuciones de supervivencia para los distintos niveles de LOC_COMB_VS.

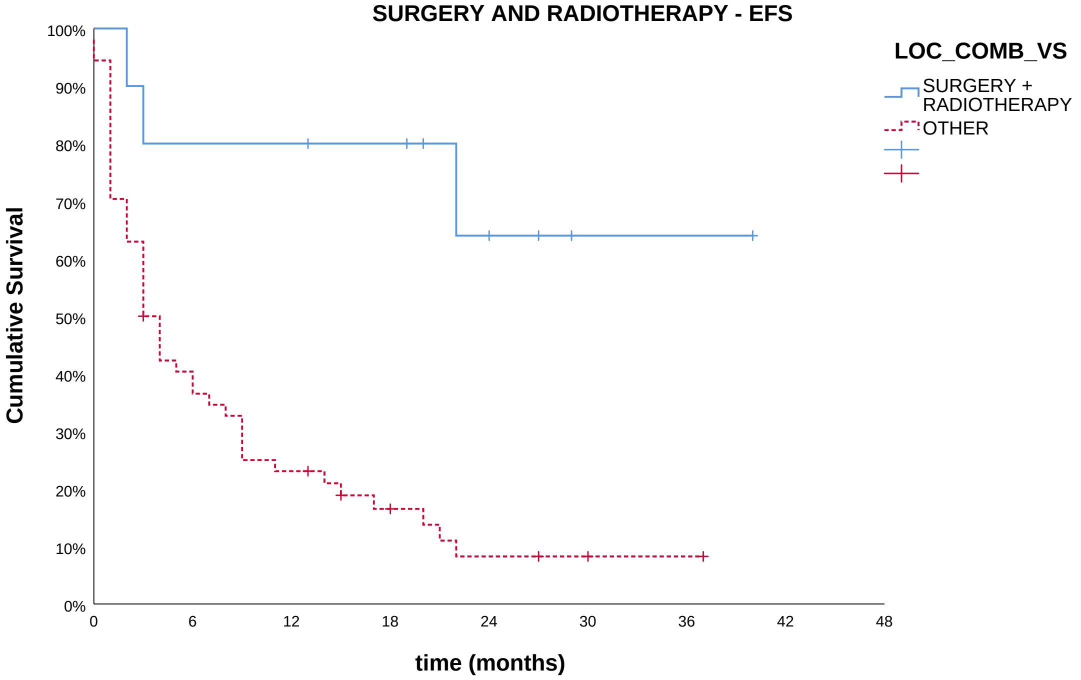

Análisis de supervivencia

Variable de supervivencia : SLE (meses, episodios)

Tabla de mortalidad

Controles de primer orden		Hora de inicio del intervalo	Número que entra en el intervalo
LOC_COMB_VS	CIRUGÍA + RADIOTERAPIA	0	9
		6	7
		12	7
		18	6
		24	4
		30	1
		36	1
	RESTO	0	53
		6	20
		12	11
		18	6
		24	3
		30	2
		36	1

Tabla de mortalidad

Controles de primer orden		Hora de inicio del intervalo	Número de retirada durante el intervalo
LOC_COMB_VS	CIRUGÍA + RADIOTERAPIA	0	0
		6	0
		12	1
		18	2
		24	3
		30	0
		36	1
	RESTO	0	1
		6	0
		12	2
		18	1
		24	1
		30	1
		36	1

Tabla de mortalidad

Controles de primer orden		Hora de inicio del intervalo	Número expuesto a riesgo
LOC_COMB_VS	CIRUGÍA + RADIOTERAPIA	0	9,000
		6	7,000
		12	6,500
		18	5,000
		24	2,500
		30	1,000
		36	,500
	RESTO	0	52,500
		6	20,000
		12	10,000
		18	5,500
		24	2,500
		30	1,500
		36	,500

Tabla de mortalidad

Controles de primer orden		Hora de inicio del intervalo	Número de eventos terminales
LOC_COMB_VS	CIRUGÍA + RADIOTERAPIA	0	2
		6	0
		12	0
		18	0
		24	0
		30	0
		36	0
	RESTO	0	32
		6	9
		12	3
		18	2
		24	0
		30	0
		36	0

Tabla de mortalidad

Controles de primer orden		Hora de inicio del intervalo	Proporción que termina
LOC_COMB_VS	CIRUGÍA + RADIOTERAPIA	0	,22
		6	,00
		12	,00
		18	,00
		24	,00
		30	,00
		36	,00
	RESTO	0	,61
		6	,45
		12	,30
		18	,36
		24	,00
		30	,00
		36	,00

Tabla de mortalidad

Controles de primer orden		Hora de inicio del intervalo	Proporción que sobrevive
LOC_COMB_VS	CIRUGÍA + RADIOTERAPIA	0	,78
		6	1,00
		12	1,00
		18	1,00
		24	1,00
		30	1,00
		36	1,00
	RESTO	0	,39
		6	,55
		12	,70
		18	,64
		24	1,00
		30	1,00
		36	1,00

Tabla de mortalidad

Controles de primer orden		Hora de inicio del intervalo	Proporción acumulada que sobrevive al final del intervalo
LOC_COMB_VS	CIRUGÍA + RADIOTERAPIA	0	,78
		6	,78
		12	,78
		18	,78
		24	,78
		30	,78
		36	,78
	RESTO	0	,39
		6	,21
		12	,15
		18	,10
		24	,10
		30	,10
		36	,10

Tabla de mortalidad

Controles de primer orden		Hora de inicio del intervalo	Error estándar de la proporción acumulada que perdura al final del intervalo
LOC_COMB_VS	CIRUGÍA + RADIOTERAPIA	0	,14
		6	,14
		12	,14
		18	,14
		24	,14
		30	,14
		36	,14
	RESTO	0	,07
		6	,06
		12	,05
		18	,04
		24	,04
		30	,04
		36	,04

Tabla de mortalidad

Controles de primer orden		Hora de inicio del intervalo	Densidad de probabilidad
LOC_COMB_VS	CIRUGÍA + RADIOTERAPIA	0	,037
		6	,000
		12	,000
		18	,000
		24	,000
		30	,000
		36	,000
	RESTO	0	,102
		6	,029
		12	,011
		18	,009
		24	,000
		30	,000
		36	,000

Tabla de mortalidad

Controles de primer orden		Hora de inicio del intervalo	Error estándar de la densidad de probabilidad
LOC_COMB_VS	CIRUGÍA + RADIOTERAPIA	0	,023
		6	,000
		12	,000
		18	,000
		24	,000
		30	,000
		36	,000
	RESTO	0	,011
		6	,009
		12	,006
		18	,006
		24	,000
		30	,000
		36	,000

Tabla de mortalidad

Controles de primer orden		Hora de inicio del intervalo	Índice de riesgo
LOC_COMB_VS	CIRUGÍA + RADIOTERAPIA	0	,04
		6	,00
		12	,00
		18	,00
		24	,00
		30	,00
		36	,00
	RESTO	0	,15
		6	,10
		12	,06
		18	,07
		24	,00
		30	,00
		36	,00

Tabla de mortalidad

Controles de primer orden		Hora de inicio del intervalo	Error estándar del índice de riesgo
LOC_COMB_VS	CIRUGÍA + RADIOTERAPIA	0	,03
		6	,00
		12	,00
		18	,00
		24	,00
		30	,00
		36	,00
	RESTO	0	,02
		6	,03
		12	,03
		18	,05
		24	,00
		30	,00
		36	,00

Kaplan-Meier OS

ONLY CASES WITH LOCAL TREATMENT: SURGERY +RADIOTHERAPY VS 1 MODALITY (SURGERY OR RADIOTHERAPY)

Resumen de procesamiento de casos

LOC_COMB_VS	N total	N de eventos	Censurado	
			N	Porcentaje
CIRUGÍA + RADIOTERAPIA	10	3	7	70,0%
RESTO	20	14	6	30,0%
Global	30	17	13	43,3%

Medias y medianas para el tiempo de supervivencia

LOC_COMB_VS	Media[a]				Mediana
			Intervalo de confianza de 95 %		
	Estimación	Desv. Error	Límite inferior	Límite superior	Estimación
CIRUGÍA + RADIOTERAPIA	30,000	4,565	21,052	38,948	33,000
RESTO	18,370	3,781	10,960	25,780	9,000
Global	23,026	3,324	16,511	29,540	13,000

Medias y medianas para el tiempo de supervivencia

LOC_COMB_VS	Mediana		
		Intervalo de confianza de 95 %	
	Desv. Error	Límite inferior	Límite superior
CIRUGÍA + RADIOTERAPIA	20,288	,000	72,764
RESTO	1,112	6,820	11,180
Global	9,705	,000	32,021

a. La estimación está limitada al tiempo de supervivencia más largo, si está censurado.

Comparaciones globales

	Chi-cuadrado	gl	Sig.
Log Rank (Mantel-Cox)	3,492	1	,062

Prueba de igualdad de distribuciones de supervivencia para los distintos niveles de LOC_COMB_VS.

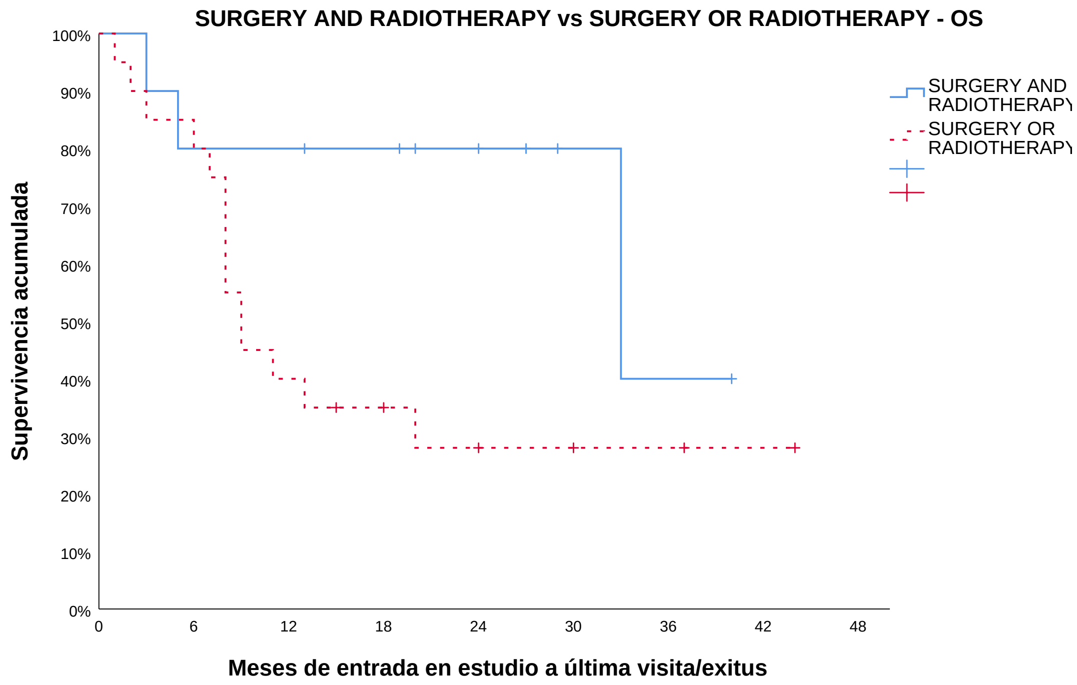

Análisis de supervivencia

Variable de supervivencia : Meses de entrada en estudio a última visita/exitus

Tabla de mortalidad

Controles de primer orden		Hora de inicio del intervalo	Número que entra en el intervalo
LOC_COMB_VS	CIRUGÍA + RADIOTERAPIA	0	10
		6	8
		12	8
		18	7
		24	5
		30	2
		36	1
	RESTO	0	20
		6	17
		12	8
		18	6
		24	4
		30	3
		36	2
		42	1

Tabla de mortalidad

Controles de primer orden		Hora de inicio del intervalo	Número de retirada durante el intervalo
LOC_COMB_VS	CIRUGÍA + RADIOTERAPIA	0	0
		6	0
		12	1
		18	2
		24	3
		30	0
		36	1
	RESTO	0	0
		6	0
		12	1
		18	1
		24	1
		30	1
		36	1
		42	1

Tabla de mortalidad

Controles de primer orden		Hora de inicio del intervalo	Número expuesto a riesgo
LOC_COMB_VS	CIRUGÍA + RADIOTERAPIA	0	10,000
		6	8,000
		12	7,500
		18	6,000
		24	3,500
		30	2,000
		36	,500
	RESTO	0	20,000
		6	17,000
		12	7,500
		18	5,500
		24	3,500
		30	2,500
		36	1,500
		42	,500

Tabla de mortalidad

Controles de primer orden		Hora de inicio del intervalo	Número de eventos terminales
LOC_COMB_VS	CIRUGÍA + RADIOTERAPIA	0	2
		6	0
		12	0
		18	0
		24	0
		30	1
		36	0
	RESTO	0	3
		6	9
		12	1
		18	1
		24	0
		30	0
		36	0
		42	0

Tabla de mortalidad

Controles de primer orden		Hora de inicio del intervalo	Proporción que termina
LOC_COMB_VS	CIRUGÍA + RADIOTERAPIA	0	,20
		6	,00
		12	,00
		18	,00
		24	,00
		30	,50
		36	,00
	RESTO	0	,15
		6	,53
		12	,13
		18	,18
		24	,00
		30	,00
		36	,00
		42	,00

Tabla de mortalidad

Controles de primer orden		Hora de inicio del intervalo	Proporción que sobrevive
LOC_COMB_VS	CIRUGÍA + RADIOTERAPIA	0	,80
		6	1,00
		12	1,00
		18	1,00
		24	1,00
		30	,50
		36	1,00
	RESTO	0	,85
		6	,47
		12	,87
		18	,82
		24	1,00
		30	1,00
		36	1,00
		42	1,00

Tabla de mortalidad

Controles de primer orden		Hora de inicio del intervalo	Proporción acumulada que sobrevive al final del intervalo
LOC_COMB_VS	CIRUGÍA + RADIOTERAPIA	0	,80
		6	,80
		12	,80
		18	,80
		24	,80
		30	,40
		36	,40
	RESTO	0	,85
		6	,40
		12	,35
		18	,28
		24	,28
		30	,28
		36	,28
		42	,28

Tabla de mortalidad

Controles de primer orden		Hora de inicio del intervalo	Error estándar de la proporción acumulada que perdura al final del intervalo
LOC_COMB_VS	CIRUGÍA + RADIOTERAPIA	0	,13
		6	,13
		12	,13
		18	,13
		24	,13
		30	,29
		36	,29
	RESTO	0	,08
		6	,11
		12	,11
		18	,10
		24	,10
		30	,10
		36	,10
		42	,10

Tabla de mortalidad

Controles de primer orden		Hora de inicio del intervalo	Densidad de probabilidad
LOC_COMB_VS	CIRUGÍA + RADIOTERAPIA	0	,033
		6	,000
		12	,000
		18	,000
		24	,000
		30	,067
		36	,000
	RESTO	0	,025
		6	,075
		12	,009
		18	,011
		24	,000
		30	,000
		36	,000
		42	,000

Tabla de mortalidad

Controles de primer orden		Hora de inicio del intervalo	Error estándar de la densidad de probabilidad
LOC_COMB_VS	CIRUGÍA + RADIOTERAPIA	0	,021
		6	,000
		12	,000
		18	,000
		24	,000
		30	,048
		36	,000
	RESTO	0	,013
		6	,019
		12	,009
		18	,010
		24	,000
		30	,000
		36	,000
		42	,000

Tabla de mortalidad

Controles de primer orden		Hora de inicio del intervalo	Índice de riesgo
LOC_COMB_VS	CIRUGÍA + RADIOTERAPIA	0	,04
		6	,00
		12	,00
		18	,00
		24	,00
		30	,11
		36	,00
	RESTO	0	,03
		6	,12
		12	,02
		18	,03
		24	,00
		30	,00
		36	,00
		42	,00

Tabla de mortalidad

Controles de primer orden		Hora de inicio del intervalo	Error estándar del índice de riesgo
LOC_COMB_VS	CIRUGÍA + RADIOTERAPIA	0	,03
		6	,00
		12	,00
		18	,00
		24	,00
		30	,10
		36	,00
	RESTO	0	,02
		6	,04
		12	,02
		18	,03
		24	,00
		30	,00
		36	,00
		42	,00

Kaplan-Meier EFS: ONLY CASES WITH LOCAL TREATMENT: SURGERY +RADIOTHERAPY VS 1 MODALITY (SURGERY OR RADIOTHERAPY)

Resumen de procesamiento de casos

LOC_COMB_VS	N total	N de eventos	Censurado	
			N	Porcentaje
CIRUGÍA + RADIOTERAPIA	10	3	7	70,0%
RESTO	20	18	2	10,0%
Global	30	21	9	30,0%

Medias y medianas para el tiempo de supervivencia

LOC_COMB_VS	Media[a]				Mediana
			Intervalo de confianza de 95 %		
	Estimación	Desv. Error	Límite inferior	Límite superior	Estimación
CIRUGÍA + RADIOTERAPIA	29,620	5,003	19,815	39,425	.
RESTO	9,200	2,384	4,528	13,872	4,000
Global	16,055	2,932	10,309	21,800	9,000

Medias y medianas para el tiempo de supervivencia

LOC_COMB_VS	Mediana		
		Intervalo de confianza de 95 %	
	Desv. Error	Límite inferior	Límite superior
CIRUGÍA + RADIOTERAPIA	.	.	.
RESTO	3,354	,000	10,574
Global	2,036	5,010	12,990

a. La estimación está limitada al tiempo de supervivencia más largo, si está censurado.

Comparaciones globales

	Chi-cuadrado	gl	Sig.
Log Rank (Mantel-Cox)	9,009	1	,003

Prueba de igualdad de distribuciones de supervivencia para los distintos niveles de LOC_COMB_VS.

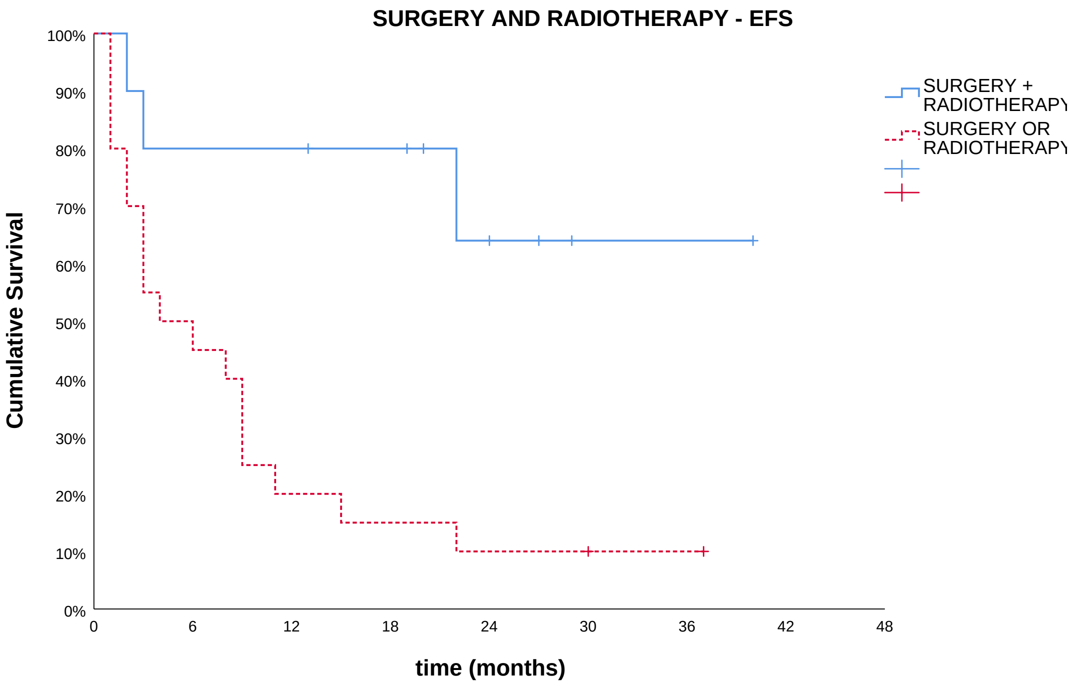

Análisis de supervivencia

Variable de supervivencia : SLE (meses, episodios)

Tabla de mortalidad

Controles de primer orden		Hora de inicio del intervalo	Número que entra en el intervalo
LOC_COMB_VS	CIRUGÍA + RADIOTERAPIA	0	9
		6	7
		12	7
		18	6
		24	4
		30	1
		36	1
	RESTO	0	20
		6	10
		12	4
		18	3
		24	2
		30	2
		36	1

Tabla de mortalidad

Controles de primer orden		Hora de inicio del intervalo	Número de retirada durante el intervalo
LOC_COMB_VS	CIRUGÍA + RADIOTERAPIA	0	0
		6	0
		12	1
		18	2
		24	3
		30	0
		36	1
	RESTO	0	0
		6	0
		12	0
		18	0
		24	0
		30	1
		36	1

Tabla de mortalidad

Controles de primer orden		Hora de inicio del intervalo	Número expuesto a riesgo
LOC_COMB_VS	CIRUGÍA + RADIOTERAPIA	0	9,000
		6	7,000
		12	6,500
		18	5,000
		24	2,500
		30	1,000
		36	,500
	RESTO	0	20,000
		6	10,000
		12	4,000
		18	3,000
		24	2,000
		30	1,500
		36	,500

Tabla de mortalidad

Controles de primer orden		Hora de inicio del intervalo	Número de eventos terminales
LOC_COMB_VS	CIRUGÍA + RADIOTERAPIA	0	2
		6	0
		12	0
		18	0
		24	0
		30	0
		36	0
	RESTO	0	10
		6	6
		12	1
		18	1
		24	0
		30	0
		36	0

Tabla de mortalidad

Controles de primer orden		Hora de inicio del intervalo	Proporción que termina
LOC_COMB_VS	CIRUGÍA + RADIOTERAPIA	0	,22
		6	,00
		12	,00
		18	,00
		24	,00
		30	,00
		36	,00
	RESTO	0	,50
		6	,60
		12	,25
		18	,33
		24	,00
		30	,00
		36	,00

Tabla de mortalidad

Controles de primer orden		Hora de inicio del intervalo	Proporción que sobrevive
LOC_COMB_VS	CIRUGÍA + RADIOTERAPIA	0	,78
		6	1,00
		12	1,00
		18	1,00
		24	1,00
		30	1,00
		36	1,00
	RESTO	0	,50
		6	,40
		12	,75
		18	,67
		24	1,00
		30	1,00
		36	1,00

Tabla de mortalidad

Controles de primer orden		Hora de inicio del intervalo	Proporción acumulada que sobrevive al final del intervalo
LOC_COMB_VS	CIRUGÍA + RADIOTERAPIA	0	,78
		6	,78
		12	,78
		18	,78
		24	,78
		30	,78
		36	,78
	RESTO	0	,50
		6	,20
		12	,15
		18	,10
		24	,10
		30	,10
		36	,10

Tabla de mortalidad

Controles de primer orden		Hora de inicio del intervalo	Error estándar de la proporción acumulada que perdura al final del intervalo
LOC_COMB_VS	CIRUGÍA + RADIOTERAPIA	0	,14
		6	,14
		12	,14
		18	,14
		24	,14
		30	,14
		36	,14
	RESTO	0	,11
		6	,09
		12	,08
		18	,07
		24	,07
		30	,07
		36	,07

Tabla de mortalidad

Controles de primer orden		Hora de inicio del intervalo	Densidad de probabilidad
LOC_COMB_VS	CIRUGÍA + RADIOTERAPIA	0	,037
		6	,000
		12	,000
		18	,000
		24	,000
		30	,000
		36	,000
	RESTO	0	,083
		6	,050
		12	,008
		18	,008
		24	,000
		30	,000
		36	,000

Tabla de mortalidad

Controles de primer orden		Hora de inicio del intervalo	Error estándar de la densidad de probabilidad
LOC_COMB_VS	CIRUGÍA + RADIOTERAPIA	0	,023
		6	,000
		12	,000
		18	,000
		24	,000
		30	,000
		36	,000
	RESTO	0	,019
		6	,017
		12	,008
		18	,008
		24	,000
		30	,000
		36	,000

Tabla de mortalidad

Controles de primer orden		Hora de inicio del intervalo	Índice de riesgo
LOC_COMB_VS	CIRUGÍA + RADIOTERAPIA	0	,04
		6	,00
		12	,00
		18	,00
		24	,00
		30	,00
		36	,00
	RESTO	0	,11
		6	,14
		12	,05
		18	,07
		24	,00
		30	,00
		36	,00

Tabla de mortalidad

Controles de primer orden		Hora de inicio del intervalo	Error estándar del índice de riesgo
LOC_COMB_VS	CIRUGÍA + RADIOTERAPIA	0	,03
		6	,00
		12	,00
		18	,00
		24	,00
		30	,00
		36	,00
	RESTO	0	,03
		6	,05
		12	,05
		18	,07
		24	,00
		30	,00
		36	,00
