# Supplementary material for: Management and outcome of children with high-risk neuroblastoma: insights from the Spanish Society of Pediatric Hematology and Oncology (SEHOP) neuroblastoma group on refractory and relapse/progressive disease
Source: Clin Transl Oncol. 2025 Feb 25;27(8):3421–31. doi: 10.1007/s12094-025-03853-w (PMC12259807; doi:10.1007/s12094-025-03853-w)
Supplement: Supplementary file 2 — Supplementary file2 (PDF 180 KB) [file 12094_2025_3853_MOESM2_ESM.pdf]

TABLE S1. List of patients' events included in the study with type of event, treatment received and type of response at the end of treatment.

Event	Type of event	Type of R/R-P	Clinical trial	Treatment received	Response (end of treatment)
1	relapse/progression	local		TVD x2, S	PD
2	relapse/progression	metastatic		TTm x2	PD
3	relapse/progression	combined		CT x2	PD
4	refractoriness	metastatic		TTm x2	SD
5	relapse/progression	metastatic		CT	PD
6	refractoriness	metastatic		CH, N	PD
7	relapse/progression	metastatic		Tm, S	PD
8	relapse/progression	metastatic		ITm	PD
9	relapse/progression	metastatic	BEACON	TTm, S	PD
10	relapse/progression	metastatic		MIBG-T	PD
11	refractoriness	metastatic		TTm x4, S, Rt	CR
12	refractoriness	metastatic	BEACON	TVD x2, TTm-Db x6, BM, S, Rt, Db x5, Ra x6	PR
13	refractoriness	metastatic		TVD x2, BM, S, Rt, Db x5	CR
14	relapse/progression	local		TVD x2	PD
15	relapse/progression	local		Tm, S, Rt	PD
16	relapse/progression	metastatic		TTm x12, Tm x3, S, Rt, Db-Ra x3	PD
17	relapse/progression	metastatic		TTm x2	PD
18	relapse/progression	metastatic		VC	PD
19	relapse/progression	metastatic		BITm x2, MIBG-T	CR
20	relapse/progression	metastatic		TVD x2, Db-ITm x4, S, BM	PD
21	refractoriness	metastatic		ITm x5, S, BM, Rt	PR
22	relapse/progression	combined		VC	PD

23	relapse/progression	combined		TVD	PD
24	refractoriness	metastatic	VERITAS	ITm x3, Th	PD
25	relapse/progression	metastatic		CT-Db x2	PD
26	relapse/progression	metastatic		Tm, Rt, MIBG-T	CR
27	relapse/progression	metastatic		ITm x2, N, Tm-O x7	PR
28	relapse/progression	metastatic		ITm-N	Unk
29	relapse/progression	metastatic	BEACON	TTm-Db x2	PD
30	relapse/progression	metastatic		CbE x3	PD
31	relapse/progression	metastatic	IDASANUTLIN	CH, Ida, Rt	PD
32	relapse/progression	metastatic	BEACON	TTm-Db x6, BM, Rt	CR
33	relapse/progression	metastatic	BEACON	TTm-Db x4, BM, Rt	CR
34	relapse/progression	metastatic	BEACON	TTm-Db x4, CT x4	PD
35	relapse/progression	metastatic	ALOCELYVIR	ITm + Rt + A	PD
36	relapse/progression	metastatic		CbE x7	SD
37	relapse/progression	metastatic	BEACON	TTm-Db x2, S	PD
38	relapse/progression	metastatic	OMBURTUMAB	ITm, S, Rt, Om	PD
39	relapse/progression	metastatic	ALOCELYVIR	ITm x2, Db, A	PD
40	relapse/progression	metastatic	NAXITAMAB	BITm x9, N	CR
41	relapse/progression	metastatic		TTm-Db x 4	SD
42	relapse/progression	metastatic	J1O-MC-JZHD	CT x4, Erb	PD
43	relapse/progression	metastatic		BITm x6, MIBG-T, Db	CR
44	relapse/progression	metastatic		BITm x6, MIBG-T, Db	CR
45	relapse/progression	combined		TTm-Db	PD
46	relapse/progression	combined		TTm	PD
47	relapse/progression	metastatic	BEACON	TTm-Db x5, MIBG-T	PD
48	relapse/progression	metastatic	BEACON	TTm-Db x5, MIBG-T, CbE, CDV, Rt	PD

49	relapse/progression	combined		Tm x2, MIBG-T, Rt	PR
50	relapse/progression	metastatic	OMBURTUMAB	S, ITm x6, Rt, Om, N, Tm	CR
51	relapse/progression	metastatic		CT, Rt	PD
52	relapse/progression	metastatic	BEACON	ITm x2, Ifo-CbE	PD
53	relapse/progression	local		CT x3, Rt, CE-Celecoxib*	CR
54	relapse/progression	metastatic		TTm x2	PD
55	relapse/progression	metastatic		BITm x10, Rt	SD
56	relapse/progression	metastatic	CRISP	ITm x2, CRISPx2	PD
57	relapse/progression	metastatic		ITm x5, Rt, MIBG-T, L	SD
58	relapse/progression	metastatic		BITm x2	PD
59	relapse/progression	local	BEACON	TTm x2, TVD x2, S	PD
60	relapse/progression	local	J1O-MC-JZHD	CT x2, Erb, BITm x3, Db-ITm x3, S, Rt, Db x5, Ra x6	CR
61	relapse/progression	metastatic		S, BITm x6, BM, Rt, CH, Db	SD
62	relapse/progression	combined	J1O-MC-JZHD	TTm x11, Db, Erb	PD
63	relapse/progression	metastatic		ITm x4, MIBG-T	PD
64	relapse/progression	metastatic		CT x9, Db-ITm	PD
65	relapse/progression	local		TVD, ITm, Rt	PD
66	relapse/progression	metastatic	NAXITAMAB	CT x19, N	SD
67	relapse/progression	metastatic		TVD x4, Rt, CT	PD

T	TOPOTECAN
Tm	TEMOZOLOMIDE
V	VINCRISTINE
D	DOXORRUBICIN

C	CYCLOFOSFAMIDE
Cb	CARBOPLATIN
E	ETOPOSIDE
I	IRINOTECAN
Ifo	IFOSFAMIDE
CH	CHEMOTHERAPY (NOS)
Ra	13cis Retinoic Acid
S	SURGERY
Rt	RADIOTHERAPY
MIBG	THERAPEUTIC 131I-mIBG
BM	BuMel
Th	THIOTEPA
B	BEVACIZUMAB
Db	DINUTUXIMAB BETA
N	NAXITAMAB
O	OLAPARIB
Om	OMBURTUMAB
Ida	IDASANUTLIN
A	ALOCELYVIR
Erb	ERBUMINE
CRISP	CRIZOTINIB-TEMSIROLIMUS
L	LORLATINIB

*oral CE as part of metronomic, Kieran like
